# Interface Energy Alignment between Lead Halide Perovskite Single Crystals and TIPS-Pentacene

**DOI:** 10.1021/acs.inorgchem.3c01482

**Published:** 2023-09-15

**Authors:** Alberto García-Fernández, Birgit Kammlander, Stefania Riva, Danilo Kühn, Sebastian Svanström, Håkan Rensmo, Ute B. Cappel

**Affiliations:** † Division of Applied Physical Chemistry, Department of Chemistry, KTH − Royal Institute of Technology, Stockholm100 44, Sweden; ‡ Division of X-ray Photon Science, Department of Physics and Astronomy, Uppsala University, Box 516,Uppsala751 20, Sweden; § Institute Methods and Instrumentation for Synchrotron Radiation Research PSISRR, Helmholtz-Zentrum Berlin für Materialien und Energie, Albert-Einstein-Straße 15, Berlin 12489, Germany

## Abstract

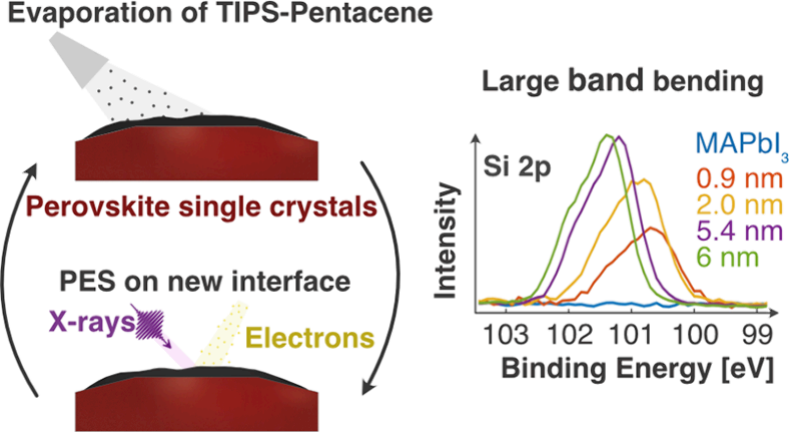

At present, there is a huge development in optoelectronic applications using lead halide perovskites. Considering that device performance is largely governed by the transport of charges across interfaces and, therefore, the interfacial electronic structure, fundamental investigations of perovskite interfaces are highly necessary. In this study, we use high-resolution soft X-ray photoelectron spectroscopy based on synchrotron radiation to explore the interfacial energetics for the molecular layer of TIPS-pentacene and lead halide perovskite single crystals. We perform ultrahigh vacuum studies on multiple thicknesses of an in situ formed interface of TIPS-pentacene with four different in situ cleaved perovskite single crystals (MAPbI_3_, MAPbBr_3_, FAPbBr_3_, and Cs_*x*_FA_1–*x*_PbBr_*y*_I_3–*y*_). Our findings reveal a substantial shift of the TIPS-pentacene energy levels toward higher binding energies with increasing thickness, while the perovskite energy levels remain largely unaffected regardless of their composition. These shifts can be interpreted as band bending in the TIPS-pentacene, and such effects should be considered when assessing the energy alignment at perovskite/organic transport material interfaces. Furthermore, we were able to follow a reorganization on the MAPbI_3_ surface with the transformation of the surface C 1s into bulk C 1s.

## Abstract 2

We investigated the interfacial energetics for a molecular layer of TIPS-pentacene and lead halide perovskite single crystals using high-resolution soft X-ray photoelectron spectroscopy based on synchrotron radiation. We performed ultrahigh vacuum studies on multiple thicknesses of an in situ formed interface of TIPS-pentacene with four different in situ cleaved perovskite single crystals (MAPbI_3_, MAPbBr_3_, FAPbBr_3_, and Cs_*x*_FA_1−*x*_PbBryI_3−*y*_).

## Introduction

Lead halide perovskites with the general formula APbX_3_, where A is usually an organic or inorganic monovalent cation and X is a halogen or a mix of halogens, have been extensively studied during the last decades due to their unprecedented multifunctional properties. Thanks to their low cost, easy tuneability, long carrier diffusion lengths, high absorption coefficients, and large charge carrier mobilities, perovskite materials have been widely used in energy and environmental applications such as solar cells, LEDs, and photodetectors.^1−4^

Perovskite-based optoelectronic devices consist of a perovskite active layer sandwiched between different selective contacts. Considering that surfaces and interfaces play an important role in their stability^5,6^ and performance, the scientific community is investing great efforts to study them.^7−11^ In this context, photoelectron spectroscopy (PES) is a widely used technique to experimentally study the electronic and chemical properties of surfaces and interfaces.^12−14^ PES provides information with elemental sensitivity and allows us to follow chemical reactions such as degradation or in situ formation of new species.^15,16^ Most of the reported experimental work is carried out on polycrystalline thin films, which do not provide clean perovskite surfaces for study, and their surface properties can vary depending on the synthesis method including, for example, unknown contaminations and solvent molecules. We recently addressed this problem by measuring in situ cleaved single crystals under UHV conditions and publishing a combined experimental and theoretical core level and valence band analysis of clean perovskite single-crystal surfaces. Such a system could be used to determine the fundamental properties of perovskite single-crystalline surfaces, and reference spectra for future photoelectron spectroscopy investigations were obtained.^17^ In the present work, we go one step further and study the interface between clean perovskite surfaces and an organic transport material. We chose a well-studied molecule that contains a specific element for the material (Si) and can be evaporated and measured in situ, providing the scientific community with a model system to contribute to the interfacial understanding of perovskite and transport materials.

Pentacene (C_22_H_14_) is a widely used organic molecule for molecular devices such as organic thin-film transistors (OTFTs), organic light emitting diodes (OLEDs), and organic thin-film sensors.^18^ Due to its structure, pentacene is sensitive to moisture, UV light, and oxygen and therefore it should preferentially be deposited by evaporation techniques, which is a disadvantage for the fabrication of large-area devices. These problems can be reduced by the addition of substituents onto the aromatic core of pentacene. In 2001, Anthony et al. reported two bis(triisopropylsilylethynyl)pentacene derivatives and studied the effect of this functionalization.^19^ Since then, 6,13-bis(triisopropylsilylethynyl)pentacene, also known as TIPS-pentacene, has become a widely used organic molecule for organic thin-film transistors and in photodetection and imaging applications.^20−23^ In addition, TIPS-pentacene is one of the most widely studied singlet fission (SF) materials due to its high SF yield and mobilities.^24−27^

In 2015, Kazim et al. fabricated an FTO|TiO_2_|MAPbI_3_|TIPS-pentacene|Au solar cell with 11.8% of efficiency (where MA is methylammonium (CH_3_NH_3_^+^)), suggesting that TIPS-pentacene could work as a hole transport material in perovskite solar cells.^28^ In 2019, Beard and co-workers coupled TIPS-pentacene derivatives to CsPbBr_3_ nanocrystals. They reported that an efficient triplet generation occurred via SF in the TIPS-pentacene molecules together with a singlet energy transfer through a Dexter-type exchange mechanism at the TIPS-pentacene|CsPbBr_3_ interface.^29^ Following this line, Lee et al.^30^ and Guo et al.^31^ studied the charge transfer dynamics in a TIPS-pentacene|MAPbI_3_ bilayer structure by several spectroscopic techniques. The different reports are contradictory in several points, and there is no clear mechanism for this transfer process. The mismatch of results could be due to different factors such as the influence of the perovskite/substrate interface, surface degradation or contamination, accurate level assignments of HOMO and LUMO of TIPS-pentacene and valence and conduction bands of perovskite, and fabrication conditions of thin films. Therefore, these reports show that detailed investigations of interfaces are needed to give a better understanding of their energy alignment.

In this work, we study the evolution of the interfacial energetics of in situ formed interfaces between TIPS-pentacene and four different in situ cleaved perovskite single crystals using synchrotron-based high-resolution soft X-ray photoelectron spectroscopy. The studied perovskite compositions are MAPbI_3_, MAPbBr_3_, FAPbBr_3_, and Cs_*x*_FA_1–*x*_PbBr_*y*_I_3–*y*_, where MA is methylammonium (CH_3_NH_3_^+^) and FA is formamidinium (CH_5_N_2_^+^). We measure the energy levels of both perovskite and TIPS-pentacene together in the same experiment under the same conditions, being able to present a detailed analysis of the interface evolution.

## Experimental Methods

### Synthesis of Materials

6,13-Bis(triisopropylsilylethynyl)pentacene (C_44_H_54_Si_2_ TIPS-pentacene) was purchased from Ossila and evaporated without any further treatment.

Single crystals of MAPbI_3_, MAPbBr_3_, FAPbBr_3_, and Cs_*x*_FA_1–*x*_PbBr_*y*_I_3–*y*_ between 0.5 and 1 cm in diameter were obtained by inverse temperature crystallization. MAI, FAI, MABr, FABr, and CsBr were purchased at Sigma-Aldrich; PbI_2_ and PbBr_3_ were purchased at TCI. All starting materials were used without any modification. AX:PbX (1:1) (A = MA/FA and X = Br/I) 1 M solutions using γ-butyrolactone (GBL) for MAPbI_3_ and dimethylformamide (DMF) for MAPbBr_3_ and FAPbBr_3_ as solvents were prepared and stirred at room temperature until all precursors were dissolved. In the case of Cs_*x*_FA_1–*x*_PbBr_*y*_I_3–*y*_, 1:1 FAI:PbI_2_ and 1:1 CsBr:PbBr_2_ 1 M solutions were prepared using DMF as the solvent, and once the precursors were dissolved, both solutions were mixed in a ratio of 1:0.1 FAPbI_3_:CsPbBr_3_ and stirred at room temperature. All final solutions were filtered through a 0.45 μm PTFE filter and transferred to an open glass vial. The vials were heated to 100 °C for MAPbI_3_ and 80 °C for the rest of the compositions. After approximately 1–2 h, selected crystals were transferred to a new solution that was already at the desired temperature depending on the composition. This process was repeated as many times as it was needed until crystals with the desired size were obtained. The best crystals were selected to be characterized by PES. The remaining crystals from the same batch were ground using a mortar and pestle and characterized by powder X-ray diffraction analysis. As can be seen in Figure S2, all synthesized materials are single-phase, without any impurity and in agreement with the single-crystal XRD profile.

Single crystals were transported to the FlexPES beamline at the MAX IV facility, located in Lund, Sweden and to the CoESCA endstation at the UE-52 PGM beamline at the BESSY II electron storage ring, located in Berlin, Germany for measurements. EPO-TEK H20E two-component epoxy was used to mount the crystals on sample plates. The sample plates were heated to 100 °C under ambient conditions for 1 h. Once the epoxy was cured, single crystals were introduced in a vacuum chamber and cleaved under a pressure of around 10^–8^ mbar. After being cleaved, samples were immediately transferred to the main chamber. All measurements were done under a pressure of around 10^–10^ mbar. TIPS-pentacene layers were evaporated sequentially on the clean single-crystal surfaces without breaking vacuum using an alumina-coated tungsten coil high-resistance homemade evaporator. Before the first evaporation, the chamber was pumped down to 10^–9^ mbar and the source was degassed to avoid exposure to contaminants.

### Photoelectron Spectroscopy Measurements and Analysis

PES measurements were carried out at the FlexPES beamline^32^ at the MAX IV facility and at the CoESCA endstation at the BESSY II electron storage ring.^33^ At FlexPES, the X-rays were generated by a linearly polarized undulator with a period length of 54.4 mm and monochromated using a plane grating monochromator (modified Zeiss SX700). The X-ray intensity was controlled by adjusting the exit slit to 10 μm for all photon energies. To minimize beam damage, a defocused beam was used. Photoelectrons were detected by a Scienta DA30-L (W) analyzer in normal emission from the samples. The energy pass was 100 eV for the core levels and 50 eV for valence band characterization. The step size was 0.05 eV in the valence band region and 0.1 eV in the core-level measurements. At the CoESCA endstation, the single-bunch X-rays were generated using a UE-52 undulator by pulse picking by resonant excitation (PPRE)^34^ and monochromated using a plane grating monochromator. The exit slit was set to 100 μm for all photon energies, and normal incidence on the sample was used. Photoelectrons were detected using a Scienta ArTOF2-EW (56° angular acceptance) spectrometer (angle-resolved time-of-flight spectrometer), mounted at an angle of 54° relative to the X-rays. With the use of the angle-resolved time-of-flight (ArTOF) spectrometers,^33^ effects of beam damage and sample charging are avoided. The high spectrometer transmission allowed measurements with a low X-ray flux, high signal intensities, and short measurement times. Perovskite compositions containing bromide showed some beam damage at the FlexPES beamline, which could be minimized at the CoESCA endstation. Despite this advantage, the CoESCA endstation presents some drawbacks such as a less exact energy calibration due to the use of an ArTOF spectrometer instead of having a hemispherical analyzer (e.g., FlexPES beamline). The energy scale of the ArTOF spectrometer was calibrated by setting the binding energy difference of I 4d and I 3d to 569.9 eV, as was successfully done in our previous work.^16^ The measurements were carried out at 535 eV. Additionally, a photon energy of 758 eV was used for measuring the time of the O 1s. At the CoESCA endstation, the signal intensities varied between different measurements, and therefore, the intensities of measurements after different evaporations could not be directly compared.

The photoelectron spectra of the core levels were fitted using a pseudo-Voigt function^35^ with a linear or Herrera-Gomez et al.^36^ background as needed. An asymmetrical peak shape was used to fit the C 1s and N 1s signals of FA-based perovskites.^37^ The binding energies were energy-calibrated by measuring the Au 4f_7/2_ core level from a grounded clean Au foil and placing it at 84.0 eV. For a comparison between data obtained at CoESCA and at FlexPES, an internal calibration against Pb 4f was used (described in the text). Inelastic mean free paths (IMFPs) for emitted electrons were estimated using the TPP-2M equation.^38,39^ To calculate the IMFP in TIPS-pentacene layers, a density of 1.10 g/cm^3^, molar weight of 639 g/mol, number of valence electrons of 238, and an optical band gap of 1.70 eV^40,41^ were used in the calculations.

### X-ray Diffraction (XRD)

XRD was measured under ambient conditions using a Siemens D5000 X-ray diffractometer using Cu Kα (λ = 1.5406 Å) radiation generated at 40 kV and 30 mA. The scans from 2 theta were collected between 10 and 45° using a step size of 0.01°.

## Result and Discussion

MAPbI_3_, MAPbBr_3_, FAPbBr_3_, and Cs_*x*_FA_1–*x*_PbBr_*y*_I_3–*y*_ single crystals were in situ cleaved in vacuum at both beamlines, and their surfaces were characterized by photoelectron spectroscopy (PES) using different photon energies (130, 535, and 758 eV). After their characterization, a TIPS-pentacene|perovskite interface was in situ formed by the evaporation of different amounts of TIPS-pentacene (C_44_H_54_Si_2_, from here referred to as TIPS-Pen). Once the desired amount of TIPS-Pen was successfully evaporated, perovskite single crystals were immediately transferred to the measurement chamber without breaking the vacuum and N 1s, C 1s, Pb 4f, Si 2p, Cs 4d, Br 3d, I 4d, and Pb 5d core levels and valence band were measured. This methodology allows us to follow the chemical changes and band alignment at interface with different TIPS-Pen thicknesses.

**Figure 1:**
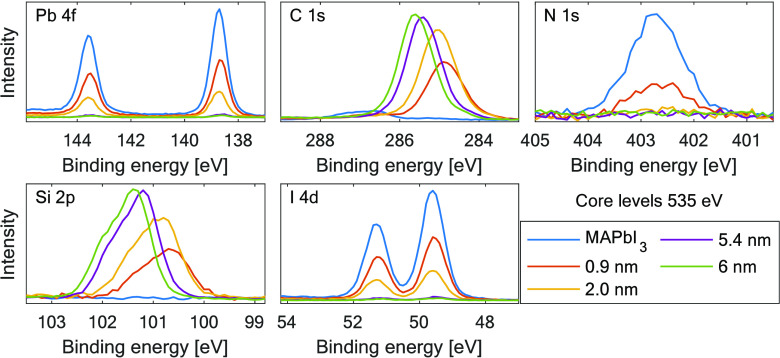
Photoelectron spectra of the Pb 4f, C 1s, N 1s, I 4d, and Si 2p core levels recorded from a MAPbI_3_ single crystal cleaved under a vacuum (blue line) and after several TIPS-Pen evaporations (other lines). All core levels were measured using the FlexPES beamline at the MAX IV synchrotron with a photon energy of 535 eV. Binding energies were energy-calibrated against Au 4f_7/2_ at 84.0 eV.

Figure 1 shows a representative example of the MAPbI_3_ results obtained at the FlexPES beamline using a photon energy of 535 eV. Additionally, the O 1s core level was recorded on the pristine perovskite and after every evaporation using a photon energy of 758 eV. As shown in Figure S3 in the Supporting Information, no O 1s signal could be detected, confirming the absence of oxygen contamination during all the experiments. To keep track of possible degradation due to X-ray damage in the MAPbI_3_ sample, we also measured C 1s and Pb 4f core levels in a different spot on the same single crystal with less X-ray exposure. Both spots showed the same results (Figure S4 in the Supporting Information).

As can be seen in Figure 1, intensities of all MAPbI_3_ core levels decrease when the amount of evaporated TIPS-Pen increases. The intensity of C 1s and N 1s perovskite core-level signals fell below the detection limits of the measurement after the second evaporation, while Pb 4f and I 4d core levels still could be detected also at the largest thicknesses. The addition of TIPS-Pen can be monitored by the appearance of the C 1s and Si 2p TIPS-Pen core levels after the first evaporation and its subsequent increase in intensity. More detailed information was obtained from curve fitting of all core levels (Figures S5 and S6, Supporting Information). Details of the fitting procedures are given in the Experimental Methods section. As an example, the Pb 4f core-level intensity decreases by 47% after the first evaporation and by 97% after the third evaporation, while the intensity of Si 2p increases by more than 200% from the first to the last evaporation. By modeling the system as a uniform two-layer system and by assuming that the X-ray intensity is constant for all measurements at one photon energy and that all core-level intensity changes therefore stem from the TIPS-Pen deposition, equations, describing how the relative core-level intensities depend on the inelastic mean free path (IMFP), can be written (see the Supporting Information for details). These equations can then be used to estimate the film thickness of the TIPS-Pen layer in calculations based on the intensity changes of the different core levels.

Based on this model, the decrease in Pb 4f and I 4d perovskite core-level intensities relative to the pristine perovskite was used to estimate the following thicknesses after each evaporation: 0.9, 2.0, 5.4, and 6.0 nm (using an average of changes in Pb 4f and I 4d core-level intensities; Table S1). In addition, two further calculations with the same model were used to double-check these values (see the Supporting Information for details). One assumes that the last evaporation measured leads to the formation of a uniformly thick layer of TIPS-Pen. This assumption is not totally correct as a very small perovskite signal is still observed after the last evaporation, but it is considered a good approximation as the C 1s signal increases by only a small amount between the last two evaporations. The intensities of the TIPS-Pen core levels in previous evaporations relative to the final one were then used to estimate the TIPS-Pen thickness (Table S1). Similar values were obtained for the thickness of the first two layers compared with the values calculated from the I 4d and Pb 4f intensities. Finally, the thickness of the first evaporated layer was also estimated from a calculation using the relative intensities of the TIPS-Pen C 1s and perovskite C 1s core levels (Table S1). All three calculations give similar results for the thickness of the first evaporated layer. While the assumption that evaporation leads to a uniform layer-by-layer growth of TIPS-Pen is unlikely to be completely correct, the agreement between the different calculations suggests that the model used and the thickness estimations are reasonable. Furthermore, the almost complete disappearance of the N 1s core-level signal after two evaporations indicates that no significant fraction of the perovskite surface remains uncovered after two evaporations.

As can be seen in Figure 1 and Figure S5, Supporting Information, the Pb 4f and I 4d core levels show one spin–orbit doublet with a full width at half-maximum (FWHM) below 0.8 eV for both core levels. No signal of Pb^0^ was detected, which would appear at 137.0 eV binding energy.^42^ The N 1s core level can also be fitted with a single peak but is wider than Pb and I core levels (FWHM ≈ 1 eV). All relative positions for the perovskite core levels match with what is described in our recent paper.^17^ After TIPS-Pen evaporation, the binding energy positions of the perovskite core levels remain similar and shift less than 0.1 eV.

**Figure 2:**
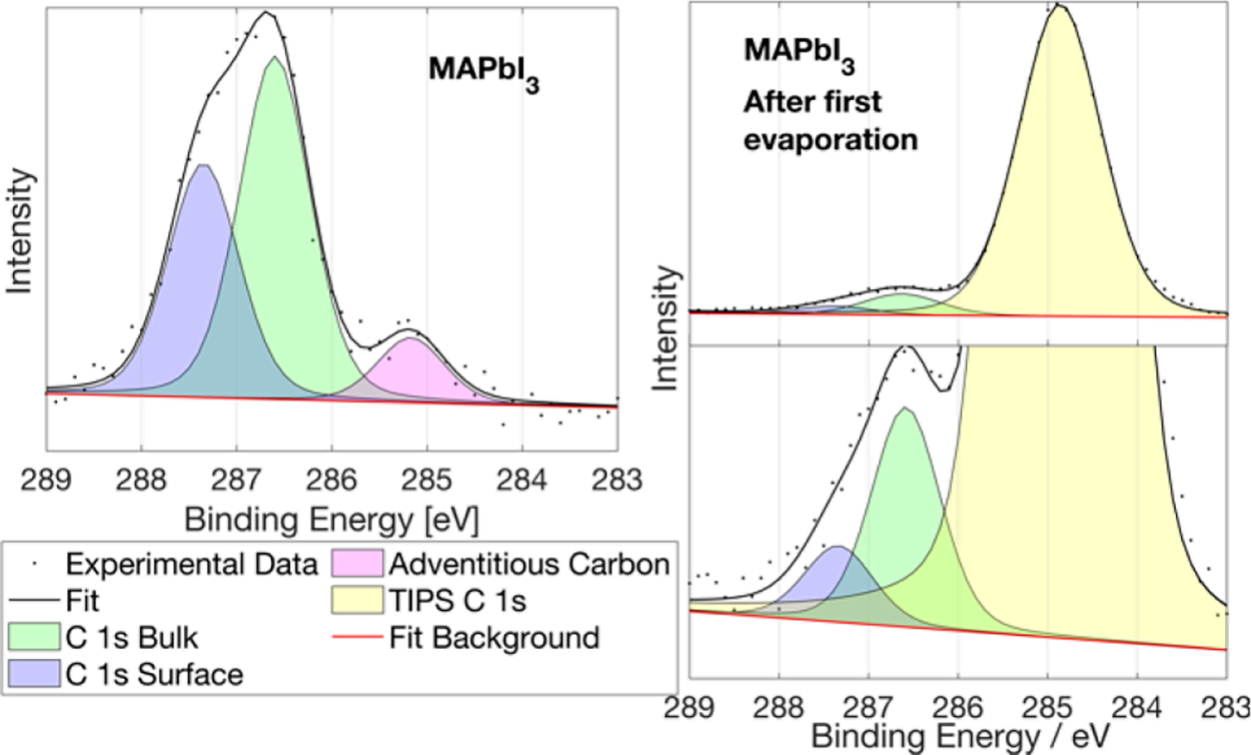
C 1s core-level spectra of pristine MAPbI_3_ single crystals (left) and after the first TIPS-Pen evaporation (right). Both spectra were measured at 535 eV and calibrated against Au 4f. Both spectra were fitted with three contributions. Two contributions related to MAPbI_3_:MA^+^ carbon toward the bulk (green) and MA^+^ carbon at the surface (blue) and one contribution related to adventitious carbon on the pristine perovskite (pink) and TIPS-Pen carbon after the first evaporation (yellow).

The C 1s core level is more complex. In agreement with our recent results, the MAPbI_3_ C 1s core level has two different contributions from the methylammonium cation (Figure 2, left), one at lower binding energies, related to the bulk, and the second one at higher binding energies, which is related to the surface carbon from the perovskite. The relation between those contributions is *C*_surf_/*C*_bulk_= 0.72, which in agreement with our previous results suggests that the surface is mostly but not fully MAI-terminated.^17^ A minor contribution of adventitious carbon around 285.0 eV can also be detected, which is minor and is expected to have limited effects on the results here presented. In this work, we not only detect both MAPbI_3_ C 1s contributions, but we are also able to follow their evolution while the TIPS-Pen|perovskite interface is forming. After the first TIPS-Pen evaporation (Figure 2, right), a new C 1s contribution from TIPS-Pen appears at a binding energy of 284.9 eV. Despite that, both C 1s contributions for the perovskite can still be resolved and deconvoluted, obtaining a *C*_surf_/*C*_bulk_ = 0.40. The change in the ratio indicates a reorganization of the MAPbI_3_ surface, where the preferential orientation of the MA surface carbon is changing and becoming more like bulk carbon with the addition of TIPS-Pen. After the second evaporation, no perovskite C 1s contribution could be detected and these effects could not be analyzed further. To follow possible chemical changes on the perovskite after the evaporations, the I 4d/Pb 4f ratio after each evaporation can also be analyzed. As the decrease in the I 4d and Pb 4f core level suggested very similar thicknesses of the TIPS-Pen layer, this suggests that the iodide to Pb ratio at the perovskite surface does not change upon evaporation and we can conclude that no chemical reactions occur upon TIPS-Pen deposition.

Focusing our attention on the TIPS-Pen core levels (C 1s and Si 2p) presented in Figure 1, we can see that their intensities increase and their peak positions are shifted to higher binding energies after each evaporation. A difference of more than 0.5 eV in the C 1s and Si 2p TIPS-Pen core-level positions was obtained by comparing the binding energies after the first and the third evaporation, suggesting a substantial shift in the energy alignment and may be interpreted as large band bending in the TIPS-Pen. Fits after the first and third evaporation are shown in Figure S6, Supporting Information. As reported by Griffith et al.,^43^ the TIPS-Pen shows a similar shift to higher binding energies on a Au substrate. This was discussed as a charging effect at a large thickness of more than 20 nm. In our study, we exclude charging effects related to the photoemission process at thicknesses below 6 nm because we observed a consistent shift to higher binding energies of TIPS-Pen core levels with increasing thickness at all measured photon energies as well as at two different beamlines (FlexPES and CoESCA) with different setups and X-ray fluxes (see the Experimental Methods section). We therefore assign the observed shifts mostly to change in the energy-level alignment upon the deposition as a consequence of a redistribution of charges, described as band bending. However, we exclude the thickest evaporated layer from the valence band analysis as the large shift observed between a thickness of 5.4 and 6.0 nm might contain charging effects.

**Figure 3:**
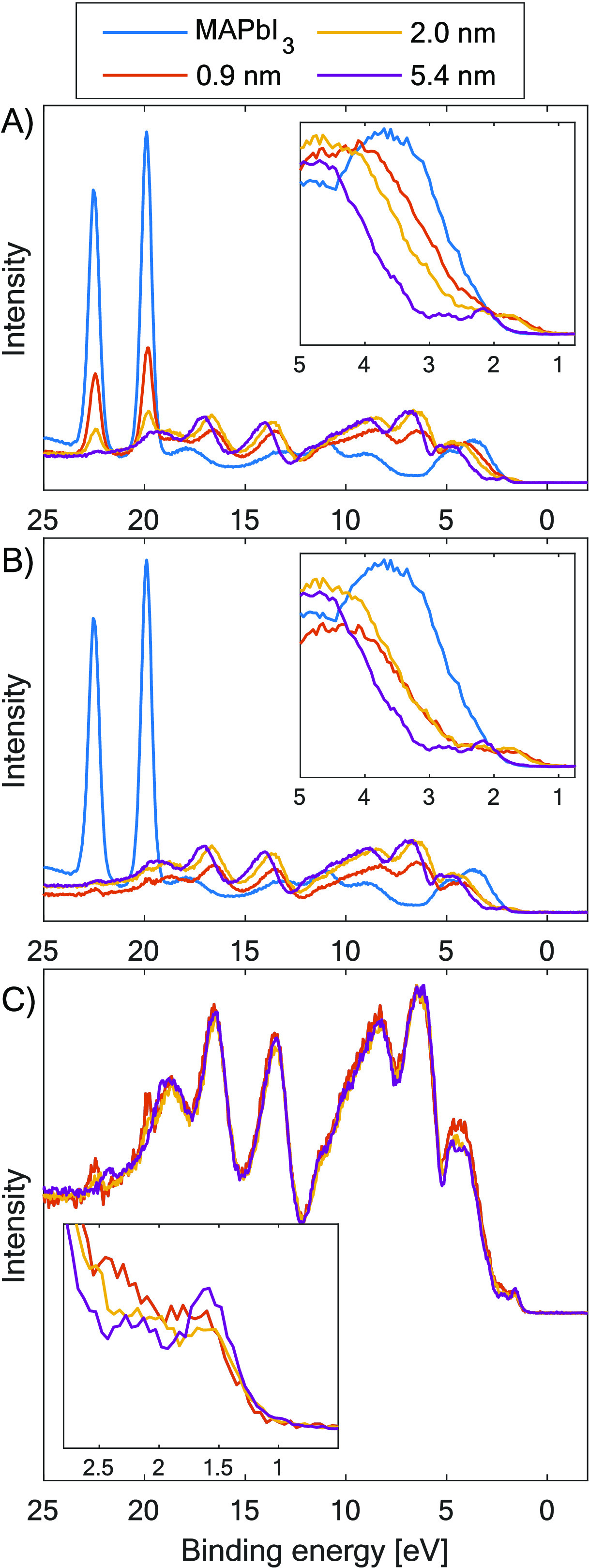
Valence band region of MAPbI_3_ (blue line) and after different TIPS-Pen evaporations (red, yellow, and purple lines) measured at 130 eV at the FlexPES beamline. (A) Full-range valence band data (zoom on the HOMO region). (B, C) Valence band spectra with perovskite contribution subtracted after the first and second evaporation (zoom on the HOMO region) and MAPbI_3_ spectrum after the third evaporation (5.4 nm), same as in panel (A). (A, B) Valence band region energy-calibrated to the Fermi level. Intensities shown are as measured. (C) Spectra are normalized to a maximum intensity of 1 and are aligned in binding energy to overlap.

To investigate the frontier electronic structure more directly at the interface, the valence band region was characterized by using a photon energy of 130 eV. Figure 3a shows the valence band region of MAPbI_3_ pristine perovskite (blue line) and after each TIPS-Pen evaporation (red, yellow, and purple lines). It can be seen how the Pb 5d core level related to the MAPbI_3_ perovskite (19.9 eV binding energy) decreases in intensity after the different evaporations maintaining the same position within 0.09 eV. By normalizing and calibrating the pristine perovskite spectrum to the Pb 5d intensity and position of the spectra after evaporation, we can remove the perovskite contribution from the valence band spectra by subtracting the spectrum of the pristine perovskite. Figure 3b shows the same region but after the subtraction of the perovskite contribution (blue line) to each TIPS-Pen evaporation, with the energy calibration versus the Fermi level. The highest occupied molecular orbital (HOMO) and the core levels of TIPS-Pen show similar shifts after each evaporation, confirming a substantial band bending in the TIPS-Pen. After the first evaporation, the HOMO of TIPS-Pen can be observed above the MAPbI_3_ valence band edge (Figure 3b). After the third evaporation (5.4 nm), the HOMO of TIPS-Pen is shifted to higher binding energies close to the valence band edge of the perovskite.

To facilitate a comparison of the TIPS-Pen contribution to the valence band, the spectra in Figure 3c show only the TIPS-Pen contribution to the valence band region normalized to the first evaporation and shifted in energy to overlap. As can be seen, all TIPS-Pen contributions to the valence band after each evaporation are generally rather similar regardless of the thickness of the layer, confirming the success of evaporating TIPS-Pen. However, small changes are observed in the structure of the HOMO levels (1–2.5 eV) indicating specific interactions between the molecule and the single-crystal substrate. This is further supported by a broadening of the C 1s and Si 2p peaks of the TIPS-Pen at low thickness compared to the thicker layers, showing values of 0.78 eV (Si 2p) and 1.07 eV (C 1s) for the first evaporation (0.9 nm), 0.77 eV (Si 2p) and 1.03 eV (C 1s) for the second evaporation (2 nm), and 0.68 eV (Si 2p) and 1.00 eV (C 1s) for the third and last evaporations (5.4 and 6 nm. respectively).

**Figure 4:**
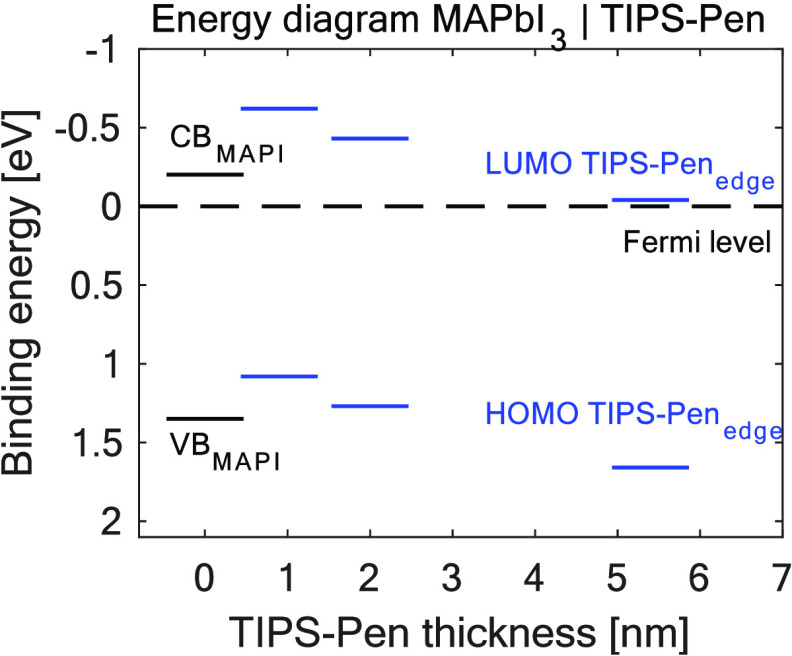
Energy-level diagram including the MAPbI_3_ valence band and conduction band (black lines) and summarizing the TIPS-Pen HOMO and LUMO band bending obtained by Si 2p shifts. The dashed line represents the Fermi level. Blue lines present the data obtained by fitting the TIPS-Pen HOMO edge.

To follow the band alignment evolution with different thicknesses of TIPS-Pen, an energy diagram at the TIPS-Pen|MAPbI_3_ interface was constructed using the data from Figure 1 and is presented in Figure 4. The MAPbI_3_ valence edge of the pristine single crystal (blue line; Figure 3) was fitted using a logarithmic fit (Figure S7, Supporting Information), obtaining a value of 1.35 eV versus the Fermi level for the MAPbI_3_ valence band (VB) edge position (bottom black line; Figure 4), a value that is in agreement with the literature.^44^ The binding energy of the TIPS-Pen HOMO edge was determined using the data from the third evaporation (purple line; Figure 3). A value of 1.89 eV for the TIPS-Pen HOMO position was obtained by a linear fit of the HOMO edge (Figure S8), which is a method commonly used to determine the HOMO position of TIPS-Pen.^43,45,46^

The MAPbI_3_ conduction band (CB) edge and TIPS-Pen lowest unoccupied molecular orbital (LUMO) binding energies were calculated by using the materials’ reported optical band gaps (1.55 eV for MAPbI_3_^47^ and 1.70 eV for TIPS-Pen^40,41^). Our estimation of the HOMO–LUMO gap in the TIPS-pentacene is based on the optical gap value. This band gap is lower than the electronic band gap by the excitonic binding energy (typically around 0.3–0.5 eV) exhibited by organic molecules.^48,49^ The MAPbI_3_ conduction band (CB) is placed 0.34 eV above the Fermi level, showing the n-type behavior of MAPbI_3_. The TIPS-Pen LUMO (third evaporation) is placed 0.04 eV above the Fermi level. The increased electronic band gap would shift the LUMO level of TIPS-Pen to lower binding energies further away from the Fermi level compared with how it is represented in Figure 4.

A combination of the core level and the valence band measurements described above was used to estimate the band alignment at the interface after each evaporation. If one assumes that the deposition of the molecular layer does not give rise to any specific material changes, then the shifts between the VB and core levels are expected to be constant. Therefore, as further described by Kraut et al.,^50^ the binding energy of the VBM/HOMO can be estimated from their binding energy difference with the core levels. C 1s and Si 2p core-level positions after each evaporation were obtained through fitting, and their individual shift was calculated using the third evaporation as a starting point. Under the assumption that the distance HOMO TIPS-Pen core level is constant after each evaporation and will not be affected by any doping or by the position of the Fermi level, the TIPS-Pen HOMO position of each evaporation was calculated using the difference obtained by the core level fitting. Both the TIPS-Pen C 1s and Si 2p core levels gave the same results within 0.03 eV. Figure 4 shows a scheme with the data obtained from the Si 2p TIPS-Pen core-level shift, and a comparison with C 1s shifts can be seen in Figure S9.

Our results show how the HOMO level of a thin layer of TIPS-Pen has a lower binding energy compared to the binding energy of the HOMO level of a thicker layer (Figure 4). Therefore, the photogenerated holes may be trapped in the interfacial regions where TIPS-Pen molecules are in contact with MAPbI_3_. These results suggest that TIPS-Pen is not a good hole transport material in a PV device when used together with a MAPbI_3_ single crystal. In the case of very thin layers of TIPS-Pen, the holes could potentially be extracted from the perovskite.

The opposite behavior can be found if we focus on the TIPS-Pen conduction band. At smaller thicknesses, TIPS-Pen will act as an electron blocker, but with larger thicknesses, the LUMO of TIPS-Pen places below the conduction band of MAPbI_3_ enabling the possibility of electron transfer from the perovskite to the TIPS-Pen.

Our results demonstrate how the energy levels of TIPS-Pen vary when comparing the layer directly adsorbed on the perovskite with different thicknesses of multilayers. Since we did not observe any chemical changes, the variations in the observed binding energies are a direct result of the redistribution of charges at the interface. This can be explained by the TIPS-Pen film giving electrons to the MAPbI_3_ perovskite. An undoped organic layer is expected to have a low carrier density, and charge transfer can therefore lead to a large change in the energy levels. On the other hand, we do not observe a significant shift in the perovskite core levels upon interface formation. This suggests that the perovskite is able to compensate for the charge redistribution across the interface, either due to larger charge density or due to ion movement. Due to the dependence of the TIPS-Pen shift on the film thickness, we refer to this phenomenon as energy-level bending in the molecular layer, as discussed in papers such as refs (51 and 52). Alternatively, in the research area of inorganic solid-state physics, it is often referred to as band bending, which, in many inorganic cases, extends for much longer distances. The characterization of TIPS-Pen energy-level alignment and bending is crucial when understanding transfer processes including those described by Lee et al.^30^ and Guo et al.^31^ where they propose an electron transfer from TIPS-Pen to MAPbI_3_ perovskite following singlet fission processes.

We further compared the impact of the perovskite composition on the redistribution of charges at the perovskite TIPS-Pen interface. These experiments were performed at the CoESCA endstation, UE-52 PGM beamline at the BESSY II synchrotron. With the use of a high transmission spectrometer, a low X-ray flux could be used to minimize the effects of beam damage and sample charging (see the Experimental Methods section for details).

The core-level spectra of MAPbI_3_, MAPbBr_3_, FAPbBr_3_, and Cs_*x*_FA_1–*x*_PbBr_*y*_I_3–*y*_ single crystals obtained at the CoESCA beamline using 535 eV photon energy are presented in Figure S10 in the Supporting Information. Additionally, the O 1s core level was recorded on the pristine perovskite, and after every evaporation using 758 eV photon energy, no O 1s signal could be detected, confirming the absence of oxygen contamination during all the experiments. It is worth noting that MAPbBr_3_ showed the formation of some Pb^0^ due to X-ray exposure with shifts in the perovskite core levels of around 0.2 eV.

The absolute signal intensity was not constant between different measurements at the CoESCA endstation, and we were therefore not able to estimate the thickness after each deposition. Furthermore, a different ratio between the C 1s and Pb 4f intensities for the pristine perovskites was obtained at CoESCA and at FlexPES (because of different measurement geometries and spectrometers, see the Experimental Methods section). We therefore used the TIPS-Pen C 1s to perovskite Pb 4f intensity ratio as a proxy for the thickness to compare the results obtained at different beamlines: The C 1s TIPS-Pen area was calculated after each evaporation and divided by the Pb 4f area from the same measurement. Considering that the ratio of C/Pb in a pristine perovskite single crystal is the same at both synchrotrons and should be equal to 1, the C 1s TIPS-Pen/Pb 4f perovskite ratio is normalized by the C 1s/Pb 4f pristine perovskite intensity ratio, obtaining a C 1s TIPS-Pen/C 1s perovskite ratio. The ratios for all measured single crystals are presented in Table S4 and will be referred to as the TIPS-Pen/perovskite ratio.

**Figure 5:**
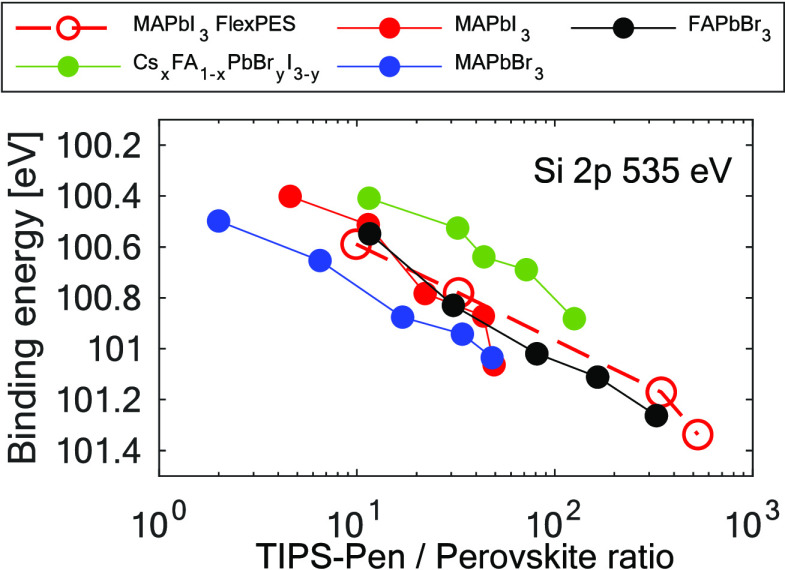
Binding energy shifts of the Si 2p TIPS-Pen core level after several evaporations on different in situ cleaved perovskite single crystals. All positions are internally calibrated against the Pb 4f core level. The dashed line represents data obtained from FlexPES beamline, and continuous lines represent data obtained at the CoESCA endstation.

Figure 5 and Figure S11 present the shifts of TIPS-Pen Si 2p and C 1s core levels, respectively, as a function of the TIPS-Pen/perovskite ratio. Binding energy positions are internally calibrated by placing the Pb 4f_7/2_ core level at 138.54 eV. TIPS-Pen core levels show a shift to higher binding energies for thicker TIPS-Pen layers regardless of the perovskite composition, indicating a downward band bending in the TIPS-Pen|perovskite interface. Our shifts show similar behavior to those reported by Griffith et al. with TIPS-Pen on the gold substrate.^43^ This suggests that upon interface formation, TIPS-Pen transfers electrons to gold in a similar way for the different perovskite compositions studied.

Moreover, Gao and co-workers used X-ray and ultraviolet photoelectron spectroscopy to study the energy-level alignment at the pentacene|MAPbI_3_ interface using thin films.^53^ In agreement with their results, we found no clear energy-level shifts, as would be expected from substantial redistribution of charges on the perovskite side. On the other hand, they report a 0.2 eV band bending to lower binding energies together with a 0.1 eV interfacial dipole shift related to the evaporated pentacene, which is in the opposite direction of the shift found by Griffith et al. on a pentacene|gold interface.^43^ In our case, we report a substantially larger redistribution of charges leading to an energy-level bending of the TIPS-Pen levels toward higher binding energies, which exceeds 0.55 eV. These results suggest that the interface between any of the studied perovskites and TIPS-Pen does not give rise to a perovskite/hole conductor interface with an electronic structure that favors efficient charge separation. To expand this outcome to other families of materials, more experiments with several thicknesses of TIPS-Pen deposited on different surfaces can be performed.

## Conclusions

We were successfully able to synthesize and in situ cleave MAPbI_3_, MAPbBr_3_, FAPbBr_3_, and Cs_*x*_FA_1–*x*_PbBr_*y*_I_3–*y*_ perovskite single crystals. Clean surfaces were characterized by using high-resolution soft X-ray photoelectron spectroscopy. We were able to evaporate several thicknesses of TIPS-Pen in situ and successfully follow the interface formation and evolution with four different perovskite compositions. The reproducibility of experiments was tested by repeating the measurements on two different beamlines.

We follow the redistribution of charges in TIPS-Pen|perovskite interfaces, which is detected by a large shift (over 0.55 eV) on TIPS-Pen energy levels. These shifts indicate that there is an electron transfer from the TIPS-Pen toward the perovskite leading to a downward band bending in the TIPS-Pen at the interface for all measured perovskite compositions. On the other hand, no shift on any perovskite core level was detected for any composition. This indicates that TIPS-Pen does not create significant band bending or new chemical bonds on the perovskite substrate. However, we were able to detect the reorganization on the MAPbI_3_ surface with the transformation of the surface C 1s into bulk C 1s.

Our results show that energy-level realignment in organic transport layers can be significant and has to be considered when designing interfaces for electronic devices with perovskite materials. Our study presents a method to analyze this realignment in situ through the use of cleaved perovskite single crystals and an evaporated transport material. This method should be used further in the future to compare interfacial charge redistribution between perovskite surfaces and promising transport materials for gaining an improved fundamental understanding of the interaction between perovskites and transport materials. While the specific results show why TIPS-Pen is not a good hole conductor in perovskite solar cells, any further studies of the interfacial charge dynamics of the perovskite/TIPS-Pen system should be motivated by means that affect the energy matching and bending, e.g., by controlled surface modification of the perovskite or by the addition of dipole molecules shifting the relative matching between the perovskite and TIPS-Pen.

## Supporting Information Available

Estimation of TIPS-Pen thickness; powder XRD patterns; photoelectron spectra of the MAPbI_3_ O 1s core level; Pb 4f and C 1s MAPbI_3_ core levels on a spot with low X-ray exposure time; fit of Pb 4f, N 1s, I 4d, and C 1s core-level spectra for MAPbI_3_. Si 2p and C 1s core-level spectra fitted for the MAPbI_3_|TIPS-Pen interface after the first and third evaporation; fit of valence band MAPbI_3_; fit of the TIPS-Pen HOMO level; energy-level diagram of the MAPbI_3_|TIPS-Pen interface; photoelectron spectra of the Pb 4f, C 1s, N 1s, I 4d, Br 3d, Cs 4d, Si 2p, and O 1s core levels from MAPbI_3_, MAPbBr_3_, FAPbBr_3_, and Cs_*x*_FA_1–*x*_PbBr_*y*_I_3–*y*_ single crystals cleaved under vacuum and after several TIPS-Pen evaporations; binding energy shifts of the C 1s TIPS-Pen core level after several evaporations on different in situ cleaved perovskite single crystals (PDF)

## References

[ref1] Wu T.; Qin Z.; Wang Y.; Wu Y.; Chen W.; Zhang S.; Cai M.; Dai S.; Zhang J.; Liu J.; Zhou Z.; Liu X.; Segawa H.; Tan H.; Tang Q.; Fang J.; Li Y.; Ding L.; Ning Z.; Qi Y.; Zhang Y.; Han L. The Main Progress of Perovskite Solar Cells in 2020–2021. Nano-micro Lett. 2021, 13, 1–18. 10.1007/s40820-021-00672-w. PMC8263824 34232444

[ref2] Rao C. N. R.; Cheetham A. K.; Thirumurugan A. Hybrid Inorganic–Organic Materials: A New Family in Condensed Matter Physics. J. Phys.: Condens. Matter 2008, 20 (8), 159801 10.1088/0953-8984/20/8/083202.

[ref3] Fang T.; Wang T.; Li X.; Dong Y.; Bai S.; Song J. Perovskite QLED with an External Quantum Efficiency of over 21% by Modulating Electronic Transport. Sci. Bull. 2021, 66 (1), 36–43. 10.1016/j.scib.2020.08.025. 36654311

[ref4] Li L.; Ye S.; Qu J.; Zhou F.; Song J.; Shen G. Recent Advances in Perovskite Photodetectors for Image Sensing. Small 2021, 2005606 10.1002/smll.202005606. 33728799

[ref5] Kammlander B.; Svanström S.; Kühn D.; Johansson F. O. L.; Sinha S.; Rensmo H.; Fernández A. G.; Cappel U. B. Thermal Degradation of Lead Halide Perovskite Surfaces. Chem. Commun. 2022, 58, 13523 10.1039/D2CC04867A. 36398706

[ref6] García-Fernández A.; Juarez-Perez E. J.; Castro-García S.; Sánchez-Andújar M.; Ono L. K.; Jiang Y.; Qi Y. Benchmarking Chemical Stability of Arbitrarily Mixed 3d Hybrid Halide Perovskites for Solar Cell Applications. Small Methods 2018, 2 (10), 1800242 10.1002/smtd.201800242.

[ref7] Li Y.; Xie H.; Lim E. L.; Hagfeldt A.; Bi D. Recent Progress of Critical Interface Engineering for Highly Efficient and Stable Perovskite Solar Cells. Adv. Energy Mater. 2022, 2102730 10.1002/aenm.202102730.

[ref8] Shao S.; Loi M. A. The Role of the Interfaces in Perovskite Solar Cells. Adv. Mater. Interfaces 2020, 1901469 10.1002/admi.201901469.

[ref9] Svanström S.; Jacobsson T. J.; Boschloo G.; Johansson E. M. J.; Rensmo H.; Cappel U. B. Degradation Mechanism of Silver Metal Deposited on Lead Halide Perovskites. ACS Appl. Mater. Interfaces 2020, 12, 7221 10.1021/acsami.9b20315. 31958007

[ref10] Luo D.; Li X.; Dumont A.; Yu H.; Lu Z. H. Recent Progress on Perovskite Surfaces and Interfaces in Optoelectronic Devices. Adv. Mater. 2021, 33, 2006004 10.1002/adma.202006004. 34145654

[ref11] Sterling C. M.; Kamal C.; Man G. J.; Nayak P. K.; Simonov K. A.; Svanström S.; García-Fernández A.; Huthwelker T.; Cappel U. B.; Butorin S. M.; Rensmo H.; Odelius M. Sensitivity of Nitrogen K-Edge X-Ray Absorption to Halide Substitution and Thermal Fluctuations in Methylammonium Lead-Halide Perovskites. J. Phys. Chem. C 2021, 125 (15), 8360 10.1021/acs.jpcc.1c02017. PMC8162417 34084262

[ref12] Schulz P.; Cahen D.; Kahn A. Halide Perovskites: Is It All about the Interfaces?. Chem. Rev. 2019, 119 (5), 3349–3417. 10.1021/acs.chemrev.8b00558. 30821958

[ref13] Béchu S.; Ralaiarisoa M.; Etcheberry A.; Schulz P. Photoemission Spectroscopy Characterization of Halide Perovskites. Adv. Energy Mater. 2020, 10 (26), 1904007–25. 10.1002/aenm.201904007.

[ref14] Svanström S.; García Fernández A.; Sloboda T.; Jacobsson T. J.; Zhang F.; Johansson F. O. L.; Kühn D.; Céolin D.; Rueff J. P.; Sun L.; Aitola K.; Rensmo H.; Cappel U. B. Direct Measurements of Interfacial Photovoltage and Band Alignment in Perovskite Solar Cells Using Hard X-Ray Photoelectron Spectroscopy. ACS Appl. Mater. Interfaces 2023, 15 (9), 12485–12494. 10.1021/acsami.2c17527. 36847773 PMC9999345

[ref15] Svanström S.; García Fernández A.; Sloboda T.; Jacobsson T. J.; Rensmo H.; Cappel U. B. X-Ray Stability and Degradation Mechanism of Lead Halide Perovskites and Lead Halides. Phys. Chem. Chem. Phys. 2021, 23 (21), 12479 10.1039/D1CP01443A. 34037011

[ref16] Svanström S.; Garciá-Fernández A.; Jacobsson T. J.; Bidermane I.; Leitner T.; Sloboda T.; Man G. J.; Boschloo G.; Johansson E. M. J.; Rensmo H.; Cappel U. B. The Complex Degradation Mechanism of Copper Electrodes on Lead Halide Perovskites. ACS Materials Au 2022, 2, 301 10.1021/acsmaterialsau.1c00038. 35578703 PMC9100662

[ref17] García-Fernández A.; Svanström S.; Sterling C. M.; Gangan A.; Erbing A.; Kamal C.; Sloboda T.; Kammlander B.; Man G. J.; Rensmo H.; Odelius M.; Cappel U. B. Experimental and Theoretical Core Level and Valence Band Analysis of Clean Perovskite Single Crystal Surfaces. Small 2022, 18 (13), 2106450 10.1002/smll.202106450. 35122466

[ref18] Yunus Y.; Mahadzir N. A.; Ansari M. N. M.; Aziz T. H. T. A.; Afdzaluddin A. M.; Anwar H.; Wang M.; Ismail A. G. Review of the Common Deposition Methods of Thin-Film Pentacene, Its Derivatives, and Their Performance. Polymers 2022, 14 (6), 1112 10.3390/POLYM14061112. 35335442 PMC8950127

[ref19] Anthony J. E.; Brooks J. S.; Eaton D. L.; Parkin S. R. Functionalized Pentacene: Improved Electronic Properties from Control of Solid-State Order [20]. J. Am. Chem. Soc. 2001, 123 (38), 9482–9483. 10.1021/JA0162459/SUPPL_FILE/JA0162459_S2.CIF. 11562247

[ref20] Montenegro Benavides C.; Biele M.; Schmidt O.; Brabec C. J.; Tedde S. F. TIPS Pentacene as a Beneficial Interlayer for Organic Photodetectors in Imaging Applications; TIPS Pentacene as a Beneficial Interlayer for Organic Photodetectors in Imaging Applications. IEEE Trans. Electron Devices 2018, 65 (4), 1516 10.1109/TED.2018.2799705.

[ref21] Kyu Park S.; Member S.; Anthony J. E.; Jackson T. N. Solution-Processed TIPS-Pentacene Organic Thin-Film-Transistor Circuits. IEEE ELECTRON DEVICE LETTERS 2007, 28, (10), , 10.1109/LED.2007.905374.

[ref22] Yu X.; Zhou N.; Han S.; Lin H.; Buchholz D. B.; Yu J.; Chang R. P. H.; Marks T. J.; Facchetti A. Flexible Spray-Coated TIPS-Pentacene Organic Thin-Filmtransistors as Ammonia Gas Sensors. J. Mater. Chem. C 2013, 1, 6532 10.1039/c3tc31412j.

[ref23] Pratap Singh A.; Jit S. Solution Processed ITO/ZnO QDs/TIPS-Pentacene/MoOx High-Performance UV-Visible Photodetector; Solution Processed ITO/ZnO QDs/TIPS-Pentacene/MoOx High-Performance UV-Visible Photodetector. IEEE Photonics Technology Letters 2022

[ref24] Herz J.; Buckup T.; Paulus F.; Engelhart J.; Bunz U. H. F.; Motzkus M. Acceleration of Singlet Fission in an Aza-Derivative of TIPS-Pentacene. 2014 10.1021/jz501102r 26277810

[ref25] Grieco C.; Doucette G. S.; Pensack R. D.; Payne M. M.; Rimshaw A.; Scholes G. D.; Anthony J. E.; Asbury J. B. Dynamic Exchange During Triplet Transport in Nanocrystalline TIPS-Pentacene Films. J. Am. Chem. Soc. 2016, 138, 16069 10.1021/jacs.6b10010. 27960344

[ref26] Wu Y.; Liu K.; Liu H.; Zhang Y.; Zhang H.; Yao J.; Fu H. Impact of Intermolecular Distance on Singlet Fission in a Series of TIPS Pentacene Compounds. J. Phys. Chem. Lett. 2014, 5, 3451–3455. 10.1021/jz5017729. 26278592

[ref27] Niu M.-S.; Yang X.-Y.; Qin C.-C.; Bi P.-Q.; Lyu C.-K.; Feng L.; Qin W.; Gao K.; Hao X.-T. Competition between Singlet Fission and Singlet Exciton Dissociation at the Interface in TIPS-Pentacene:IT-4F Blend. Org. Electron. 2019, 296 10.1016/j.orgel.2019.05.041.

[ref28] Kazim S.; Ramos F. J.; Gao P.; Nazeeruddin M. K.; Grätze M.; Ahmad S. A Dopant Free Linear Acene Derivative as a Hole Transport Material for Perovskite Pigmented Solar Cells _ Enhanced Reader. Energy Environ. Sci. 2015, 8, 1816–1823. 10.1039/C5EE00599J.

[ref29] Lu H.; Chen X.; Anthony J. E.; Johnson J. C.; Beard M. C. Sensitizing Singlet Fission with Perovskite Nanocrystals. J. Am. Chem. Soc. 2019, 141 (12), 4919–4927. 10.1021/jacs.8b13562. 30821456

[ref30] Lee S.; Hwang D.; Jung S. I.; Kim D. Electron Transfer from Triplet State of TIPS-Pentacene Generated by Singlet Fission Processes to CH3NH3PbI3 Perovskite. J. Phys. Chem. Lett. 2017, 8 (4), 884–888. 10.1021/acs.jpclett.7b00072. 28169550

[ref31] Guo D.; Ma L.; Zhou Z.; Lin D.; Wang C.; Zhao X.; Zhang F.; Zhang J.; Nie Z. Charge Transfer Dynamics in a Singlet Fission Organic Molecule and Organometal Perovskite Bilayer Structure. J. Mater. Chem. A 2020, 8 (11), 5572–5579. 10.1039/C9TA11022D.

[ref32] Preobrajenski A.; Generalov A.; Öhrwall G.; Tchaplyguine M.; Tarawneh H.; Appelfeller S.; Frampton E.; Walsh N. FlexPES: A Versatile Soft X-Ray Beamline at MAX IV Laboratory. J. Synchrotron Radiat. 2023, 30 (4), 831–840. 10.1107/S1600577523003429. 37159290 PMC10325024

[ref33] Leitner T.; Born A.; Bidermane I.; Ovsyannikov R.; Johansson F. O. L.; Sassa Y.; Föhlisch A.; Lindblad A.; Schumann F. O.; Svensson S.; Mårtensson N. The CoESCA Station at BESSY: Auger Electron–Photoelectron Coincidences from Surfaces Demonstrated for Ag MNN. J. Electron Spectrosc. Relat. Phenom. 2021, 250, 147075 10.1016/j.elspec.2021.147075.

[ref34] Holldack K.; Ovsyannikov R.; Kuske P.; Müller R.; Schälicke A.; Scheer M.; Gorgoi M.; Kühn D.; Leitner T.; Svensson S.; Mårtensson N.; Föhlisch A. Single Bunch X-Ray Pulses on Demand from a Multi-Bunch Synchrotron Radiation Source. Nat. Commun. 2014, 4010 10.1038/ncomms5010. 24874099

[ref35] Ida T.; Ando M.; Toraya H. Extended Pseudo-Voigt Function for Approximating the Voigt Profile. J. Appl. Crystallogr. 2000, 33 (6), 1311–1316. 10.1107/S0021889800010219.

[ref36] Herrera-Gomez A.; Bravo-Sanchez M.; Aguirre-Tostado F. S.; Vazquez-Lepe M. O. The Slope-Background for the near-Peak Regimen of Photoemission Spectra. J. Electron Spectrosc. Relat. Phenom. 2013, 189, 76–80. 10.1016/j.elspec.2013.07.006.

[ref37] Schmid M.; Steinrück H. P.; Gottfried J. M. A New Asymmetric Pseudo-Voigt Function for More Efficient Fitting of XPS Lines. Surf. Interface Anal. 2014, 46 (8), 505–511. 10.1002/sia.5521.

[ref38] Tanuma S.; Powell C. J.; Penn D. R. Electron Inelastic Mean Free Paths. 5. Data for 14 Organic-Compunds over the 50–2000 EV Range. Surf. Interface Anal. 1994, 21 (September 1993), 165–176. 10.1002/sia.740210302.

[ref39] Tanuma S.; Powell C. J.; Penn D. R. Calculation of Electron Inelastic Mean Free Paths (IMFPs) VII. Reliability of the TPP-2M IMFP Predictive Equation. Surf. Interface Anal. 2003, 35, 268–275. 10.1002/sia.1526.

[ref40] Davis R. J.; Lloyd M. T.; Ferreira S. R.; Bruzek M. J.; Watkins S. E.; Lindell L.; Sehati P.; Fahlman M.; Anthony J. E.; Hsu J. W. P. Determination of Energy Level Alignment at Interfaces of Hybrid and Organic Solar Cells under Ambient Environment. J. Mater. Chem. 2011, 21 (6), 1721–1729. 10.1039/C0JM02349C.

[ref41] Kadri D. A.; Karim D. A.; Seck M.; Diouma K.; Marcel P. Optimization of 6,13Bis(Triisopropylsilylethynyl)Pentacene (TIPS-Pentacene) Organic Field Effect Transistor: Annealing Temperature and Solvent Effects. Mater. Sci. Appl. 2018, 09 (11), 900–912. 10.4236/msa.2018.911065.

[ref42] Philippe B.; Park B. W.; Lindblad R.; Oscarsson J.; Ahmadi S.; Johansson E. M. J.; Rensmo H. Chemical and Electronic Structure Characterization of Lead Halide Perovskites and Stability Behavior under Different Exposures-A Photoelectron Spectroscopy Investigation. Chem. Mater. 2015, 27 (5), 1720–1731. 10.1021/acs.chemmater.5b00348.

[ref43] Griffith O. L.; Anthony J. E.; Jones A. G.; Lichtenberger D. L. Electronic Properties of Pentacene versus Triisopropylsilylethynyl- Substituted Pentacene: Environment-Dependent Effects of the Silyl Substituent. J. Am. Chem. Soc. 2010, 132 (2), 580–586. 10.1021/ja906917r. 20000766

[ref44] Schulz P.; Edri E.; Kirmayer S.; Hodes G.; Cahen D.; Kahn A. Interface Energetics in Organo-Metal Halide Perovskite-Based Photovoltaic Cells. In Energy and Environmental Science; Royal Society of Chemistry, 2014; Vol. 7, pp 1377–1381.

[ref45] Menzel D.; Al-Ashouri A.; Tejada A.; Levine I.; Guerra J. A.; Rech B.; Albrecht S.; Korte L. Field Effect Passivation in Perovskite Solar Cells by a LiF Interlayer. Adv. Energy Mater. 2022, 12 (30), 2201109 10.1002/aenm.202201109.

[ref46] Nakayama Y.; Nguyen T. L.; Ozawa Y.; Machida S.; Sato T.; Tokairin H.; Noguchi Y.; Ishii H. Complete Demonstration of the Valence Electronic Structure inside a Practical Organic Solar Cell Probed by Low Energy Photoemission. Adv. Energy Mater. 2014, 4 (7), 1301354 10.1002/aenm.201301354.

[ref47] Leguy A. M. A.; Azarhoosh P.; Alonso M. I.; Campoy-Quiles M.; Weber O. J.; Yao J.; Bryant D.; Weller M. T.; Nelson J.; Walsh A.; van Schilfgaarde M.; Barnes P. R. F. Experimental and Theoretical Optical Properties of Methylammonium Lead Halide Perovskites. Nanoscale 2016, 8, 6317–6327. 10.1039/c5nr05435d. 26477295

[ref48] Schroeder P. G.; France C. B.; Park J. B.; Parkinson B. A. Orbital Alignment and Morphology of Pentacene Deposited on Au(111) and SnS2 Studied Using Photoemission Spectroscopy. J. Phys. Chem. B 2003, 107 (10), 2253–2261. 10.1021/jp025807n.

[ref49] Martin Pope; Charles E. Swenberg. Electronic Processes in Electronic Processes in Organic Crystals and PolymersElectronic Processes in Organic Crystals and PolymersOrganic Crystals and Polymers, 2nd ed.; Oxford University press, 1999; Vol. 20.

[ref50] Kraut E. A.; Grant R. W.; Waldrop J. R.; Eowalczyk S. P. Precise Determination of the Valence Band Edge in X-Ray Photoemission Spectra: Application to Measurement of Semiconductor Interface Potentials Precise Determination of the Valence Band Edge in X-Ray Photoemission Spectra: Application to Measurement of Semiconductor Interface Potentials. Phys. Rev. Lett. 1980, 44 (24), 1620–1623. 10.1103/PhysRevLett.44.1620.

[ref51] Ishii H.; Sugiyama K.; Ito E.; Seki K. Energy Level Alignment and Interfacial Electronic Structures at Organic/Metal and Organic/Organic Interfaces. Adv. Mater. 1999, 11 (8), 605–625. 10.1002/(SICI)1521-4095(199906)11:8<605::AID-ADMA605>3.0.CO;2-Q.

[ref52] Bao Q.; Braun S.; Wang C.; Liu X.; Fahlman M. Interfaces of (Ultra)Thin Polymer Films in Organic Electronics. Adv. Mater. Interfaces 2019, 1800897 10.1002/admi.201800897.

[ref53] Ji G.; Zhao B.; Song F.; Zheng G.; Zhang X.; Shen K.; Yang Y.; Chen S.; Gao X. The Energy Level Alignment at the CH 3 NH 3 PbI 3 /Pentacene Interface. Appl. Surf. Sci. 2017, 393, 417–421. 10.1016/j.apsusc.2016.10.033.

