# Supplementary material for: Interface Energy Alignment between Lead Halide Perovskite Single Crystals and TIPS-Pentacene
Source: Inorg Chem. 2023 Sep 15;62(38):15412–20. doi: 10.1021/acs.inorgchem.3c01482 (PMC10523438; doi:10.1021/acs.inorgchem.3c01482)
Supplement: Supplementary file 1 — ic3c01482_si_001.pdf [file ic3c01482_si_001.pdf]

Supporting Information

Interface energy alignment between lead halide perovskites single crystals and TIPS-pentacene

Alberto García-Fernández,*[a] Birgit Kammlander,[a] Stefania Riva,[b] Danilo Kühn,[c] Sebastian Svanström,[b] Håkan Rensmo,*[b] Ute B. Cappel [a]

[a] Division of Applied Physical Chemistry, Department of Chemistry, KTH – Royal Institute of Technology, Stockholm, SE-100 44, Sweden.
[b] Division of X-ray Photon Science, Department of Physics and Astronomy, Uppsala University, Box 516, Uppsala, SE-751 20, Sweden
[c] Institute Methods and Instrumentation for Synchrotron Radiation Research PSISRR, Helmholtz-Zentrum Berlin für Materialien und Energie, Albert-Einstein-Straße 15, 12489 Berlin, Germany

corresponding authors email: alberto3@kth.se ; hakan.rensmo@physics.uu.se

Estimation of the TIPS-pen thickness

To estimate the thickness of the TIPS-pen layers, we model the system based on the following assumptions to describe the intensities of core level spectra from the perovskite and TIPS-pen layers:

1- The top layer of TIPS-Pen (t) is uniform and has a constant thickness (d) after each evaporation.
2- The bottom perovskite layer (b) has a thickness much greater than the probing depth of the experiment and can therefore be modelled as a layer of infinite thickness.
3- The element of which the core level is measured is uniformly distributed within one layer.
4- A single inelastic mean free path ($\boldsymbol{\lambda}$) can be used for one kinetic energy. In our case, $\boldsymbol{\lambda}$ was calculated for TIPS-Pen (details in experimental section). This treatment neglects that the mean free path might be different in the perovskite layer. However, as it is the layer thickness of the TIPS-Pen we are estimating, this effect should be small.
5- A scaling factor "A_{exp}" can be used to describe the experimental parameters which influence the intensity such as the experiment geometry, the X-ray intensity, and the detection efficiency of the spectrometer at a given kinetic energy.
6- The X-ray intensity and measurement geometry are the same for all measurements, where intensities are compared, therefore the scaling factor A_{exp} is the same for different measurements of the same core level.

The model is illustrated in Figure S1 with different thicknesses of the top layer t (TIPS-pen).

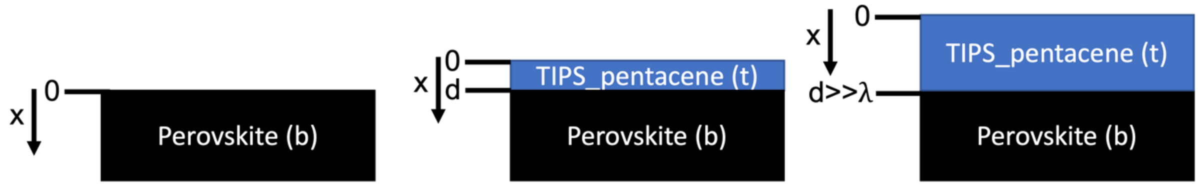

Figure S1: Illustrative pictures describing the parameters used in the following equations to calculate the TIPS-pentacene thickness.

Based on the above model, we write the following equations describing the intensity of a specific core level (j) for an element (i) at a specific photon energy for the two-layer system, where the top layer varies in thickness. The following additional parameters are used in the equations:

$N_{i,b}$ = Average atomic concentration of element "i" in the bottom layer "b".
$N_{i,t}$ = Average atomic concentration of element "i" in the top layer "t".
σ_j = Photoelectron cross section for core level "j".
λ = Inelastic mean free path as described above.
A_{exp} = Experimental scaling factor as described above.

Intensity of core level "j" from element "i" in the bottom layer "b" when d = 0 (Figure S1 left)

$$I_{b,0} = A_{\exp} N_{i,b}\sigma_j \int_0^\infty \exp\left(-\frac{x}{\lambda}\right) \mathrm{d}x = A_{\exp} N_{i,b}\sigma_j \lambda$$

Intensity of core level "j" from element "i" in the bottom layer "b" when d ≠ 0 (Figure S1 centre)

$$I_{b,d} = A_{exp}N_{i,b}\sigma_j \int_d^\infty \exp\left(-\frac{x}{\lambda}\right) \mathrm{d}x = A_{exp}N_{i,b}\sigma_j * \lambda * e^{-d/\lambda}$$

Intensity of core level "j" from element "i" in the top layer "t" when d ≠ 0 and d ≠ ∞ (Figure S1 centre)

$$I_{t,d} = A_{exp}N_{i,t}\sigma_j \int_0^d \exp\left(-\frac{x}{\lambda}\right) \mathrm{d}x = A_{exp}N_{i,t}\sigma_j * \lambda * (1 - e^{-d/\lambda})$$

Intensity of core level "j" from element "i" in the top layer "t" when d = ∞ (as d >> λ, Figure S1 right)

$$I_{t,\infty} = A_{exp}N_{i,t}\sigma_j \int_0^\infty \exp\left(-\frac{x}{\lambda}\right) d_x = A_{exp}N_{i,t}\sigma_j * \lambda$$

Using the above-described equations we can estimate the TIPS-Pen thickness by three different ways:

1) Using the decrease in Pb 4f and I 4d core levels intensity.

$$\frac{I_{b,d}}{I_{b,0}} = \frac{A_{exp}N_{i,b}\sigma_j * \lambda * e^{-d/\lambda}}{A_{exp}N_{i,b}\sigma_j * \lambda} = e^{-d/\lambda} \; ; \; \ln\left(\frac{I_{b,d}}{I_{b,0}}\right) = \ln(e^{-d/\lambda}) \; ; \ln\left(\frac{I_{b,d}}{I_{b,0}}\right) = -\frac{d}{\lambda} \; ;$$

$$d = -\lambda * \ln\left(\frac{I_{b,d}}{I_{b,0}}\right)$$

$I_{b,0}$ is determined for the measurement prior to evaporation and this calculation assumes that the X-ray intensity is the same for all measurements.

2) Considering that the last TIPS-Pen evaporation has an infinite layer thickness

$$\frac{I_{t,d}}{I_{t,\infty}} = \frac{A_{exp}N_{i,t}\sigma_j*\lambda*(1-e^{-d/\lambda})}{A_{exp}N_{i,t}\sigma_j*\lambda} = 1-e^{-d/\lambda}\ ;\ 1-\frac{I_{t,d}}{I_{t,\infty}} = e^{-d/\lambda}\ ;\ \ln\left(1-\frac{I_{t,d}}{I_{t,\infty}}\right) = -\frac{d}{\lambda}\ ;$$

$$d = -\lambda * \ln\left(1-\frac{I_{b,d}}{I_{b,0}}\right)$$

$I_{t,\infty}$ is determined for the measurement after the last evaporation and this calculation assumes that the X-ray intensity is the same for all measurements.

3) Using the relation between C 1s from perovskite and C 1s from TIPS-Pen. Considering that the perovskite C 1s cannot be detectable after the first evaporation, this method is only valid for the first layer.

$$\frac{I_{t,d}\ (\text{C 1s TIPS−Pen})}{I_{b,d}\ (\text{C 1s Perovskite})} = \frac{A_{exp}N_{i,t}\sigma_j*\lambda*(1-e^{-d/\lambda})}{A_{exp}N_{i,b}\sigma_j*\lambda*e^{-d/\lambda}} = \frac{N_{i,t}}{N_{i,b}} * \left(e^{d/\lambda}-1\right)\ ;$$

$$\frac{I_{t,d}(\text{C 1s TIPS−Pen})}{I_{b,d}(\text{C 1s Perovskite})} * \frac{N_{i,b}}{N_{i,t}} + 1 = e^{d/\lambda}\ ;\ \ln\left(\frac{I_{t,d}\ (\text{C 1s TIPS−Pen})}{I_{b,d}(\text{C 1s Perovskite})} * \frac{N_{i,b}}{N_{i,t}} + 1\right) = \frac{d}{\lambda}\ ;$$

$$\text{d} = \lambda * \ \ln\left(\frac{I_{t,d}(\text{C 1s TIPS − Pen})}{I_{b,d}(\text{C 1s Perovsktie})} * \frac{N_{i,b}}{N_{i,t}} + 1\right)$$

Results are summarized on the following table:

Table S1: Summary of results obtained with the model and equations described above.

**Thickness of TIPS-Pen layer (nm)**					
	**Decrease intensities**		**Carbon**	**Infinite thickness**	
	**Pb 4f**	**I 4d**		**C 1s**	**Si 2p**
**Evap1**	0.9	0.9	0.7	0.9	1.0
**Evap3**	2.0	2.1		2.2	2.4
**Evap5**	5.0	5.7		3.9	5.8
**Evap7**	5.6	6.3			

Values used for calculations are summarized on the following tables:

Table S2: Values used to calculate the average atomic concentration of carbon on $MAPbI_3$ and TIPS-Pentacene.

	**Density (g/cm^3)**	**Molar weight (g/mol)**	**Number of C**	**Weight (%)**	**Average atomic concentration of carbon (Density * Weight)**
**$MAPbI_3$**	4.15	618.97	1	0.019	0.080
**TIPS-Pen**	1.1	639	44	0.826	0.909

Table S3: Values of inelastic mean free path (IMFP) used for each core level.

		TIPS Values
	**Kin Energy (eV)**	**IMFP (nm)**
**Pb 4f**	396.5	1.38
**C 1s**	248.5	1
**N 1s**	132.5	0.69
**I 4d**	485.5	1.6
**Si 2p**	434	1.47

Table S4: Evaporated amount of TIPS-Pen in arbitrary units calculated with the following equation.

$$R = (\text{C 1s TIPS-Pen}_{\text{EvapX}} / \text{Pb 4f TIPS-Pen}_{\text{EvapX}}) / (\text{C 1s Perovskite} / \text{Pb 4f Perovskite})$$

	**$MAPbI_3$**		**$MAPbBr_3$**	**$CsFAPbI_3$**	**FAPbBr3**
	**FlexPES**	**CoESCA**	**CoESCA**	**CoESCA**	**CoESCA**
**Evap 1**	9.9	4.6	2.0	11.5	11.6
**Evap 2**	32.6	11.4	6.5	32.3	30.7
**Evap 3**	345.2	22.1	17.0	43.8	81.2
**Evap 4**	527.9	43.6	34.1	71.8	164.4
**Evap 5**		49.3	48.2	125.4	326.0

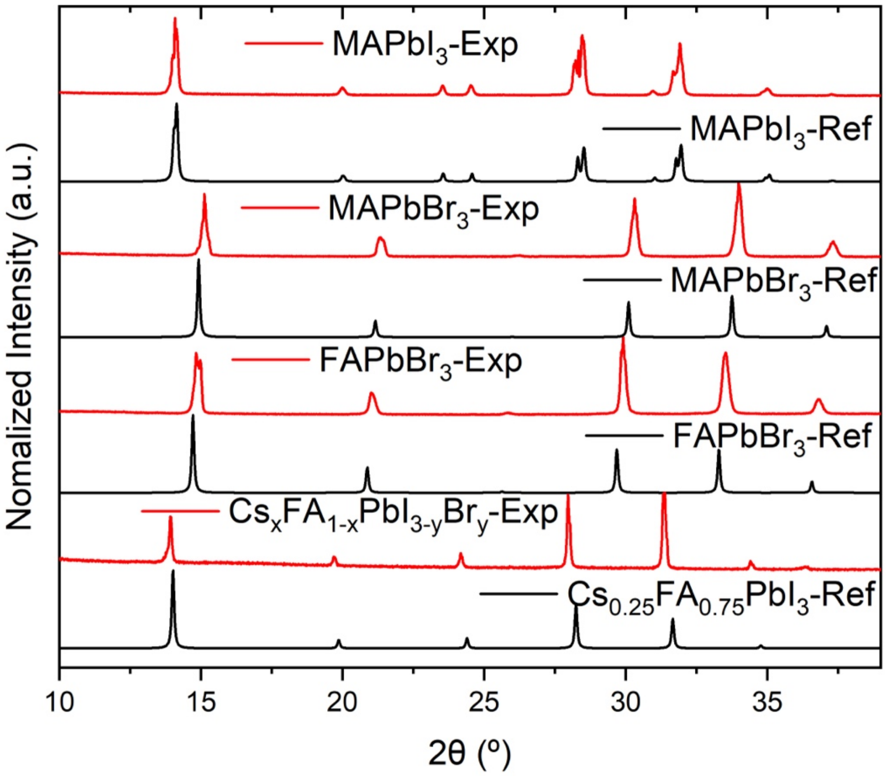

Figure S2: Experimental PXRD pattern of the polycrystalline powder of grounded single crystals (Red) of $Cs_xFA_{1-x}PbBr_yI_{3-y}$, $FAPbBr_3$, $MAPbBr_3$ and $MAPbI_3$ compared with the profile obtained from their single crystal structures at room temperature (black).

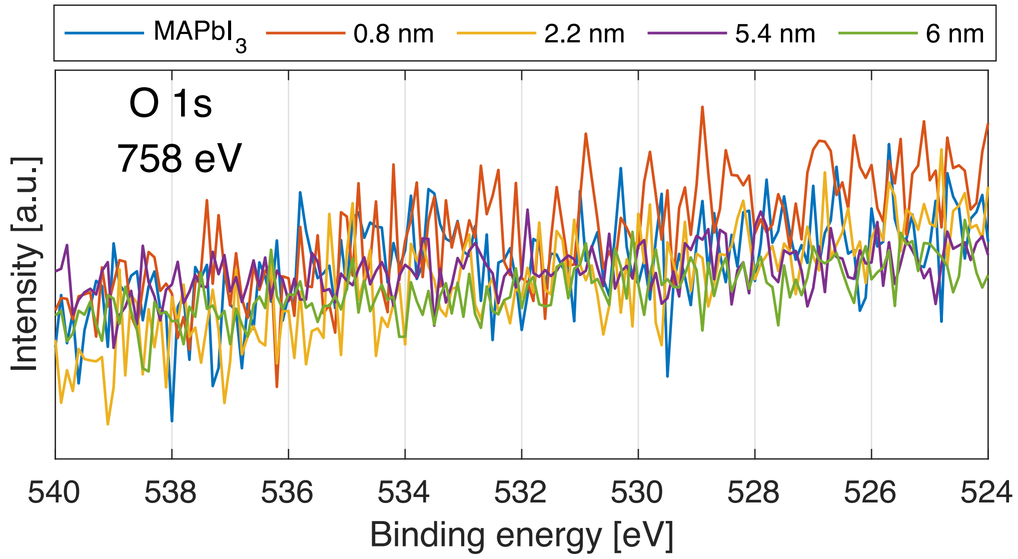

Figure S3: Photoelectron spectra of the $MAPbI_3$ O 1s core level measured using 758 eV at FlexPES beamline at MAX IV facility. Binding energies were energy calibrated against Au $4f_{7/2}$ at 84.0 eV

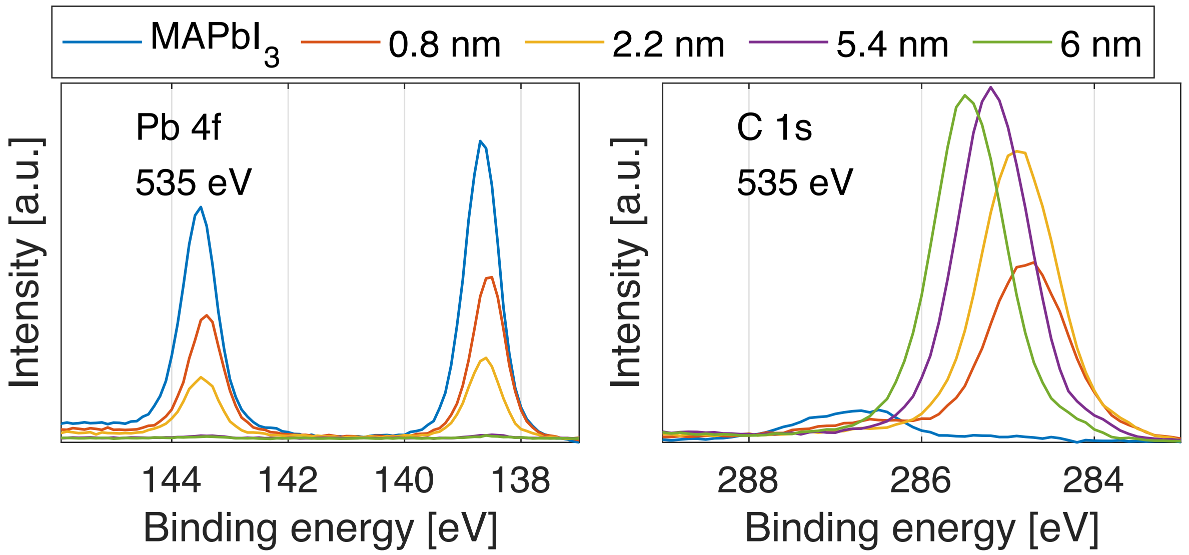

Figure S4: Pb 4f and C 1s $MAPbI_3$ core levels measured using 535 eV at FlexPES beamline on a spot with low X-ray exposure time. Binding energies were energy calibrated against Au $4f_{7/2}$ at 84.0 eV

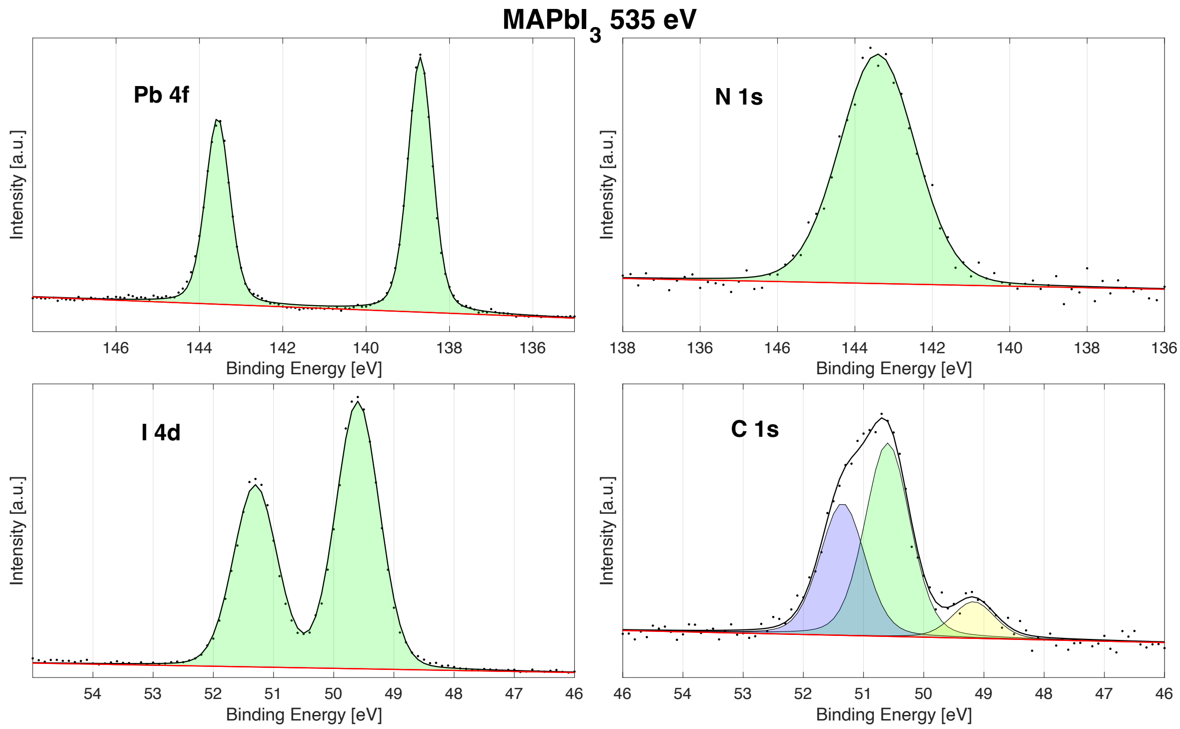

Figure S5: Pb 4f, N 1s, I 4d and C 1s core levels spectra fitted for $MAPbI_3$ single crystals cleaved under vacuum. Measured with a photon energy of 535 eV in the FlexPES beamline at MAX IV synchrotron. The binding energies were calibrated against Au $4f_{7/2}$.

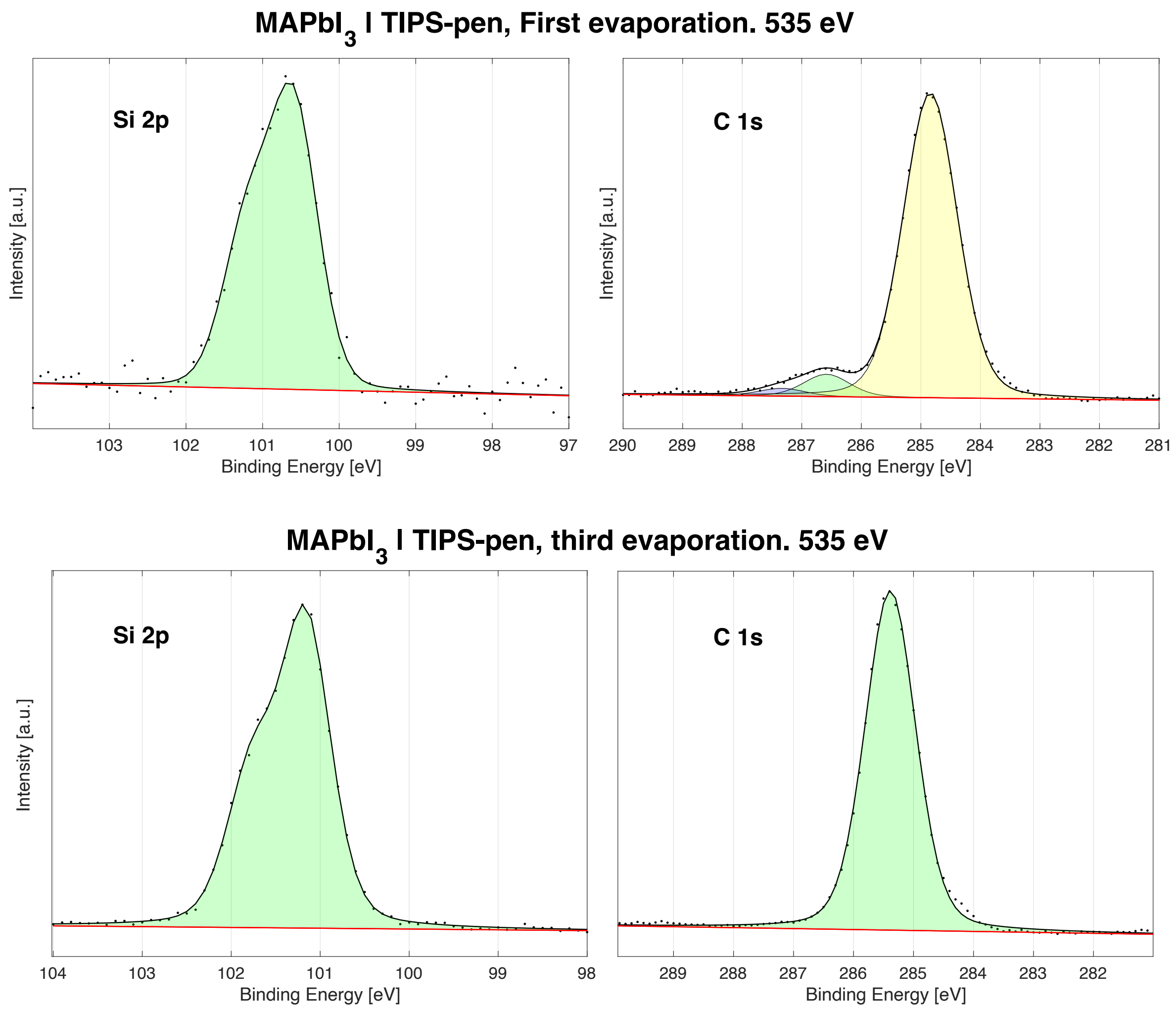

Figure S6: Si 2p and C 1s core levels spectra fitted for $MAPbI_3$ | TIPS-Pen interface after the first evaporation (top) and third evaporation (bottom). Measured with a photon energy of 535 eV in the FlexPES beamline at MAX IV synchrotron. The binding energies were calibrated against Au $4f_{7/2}$.

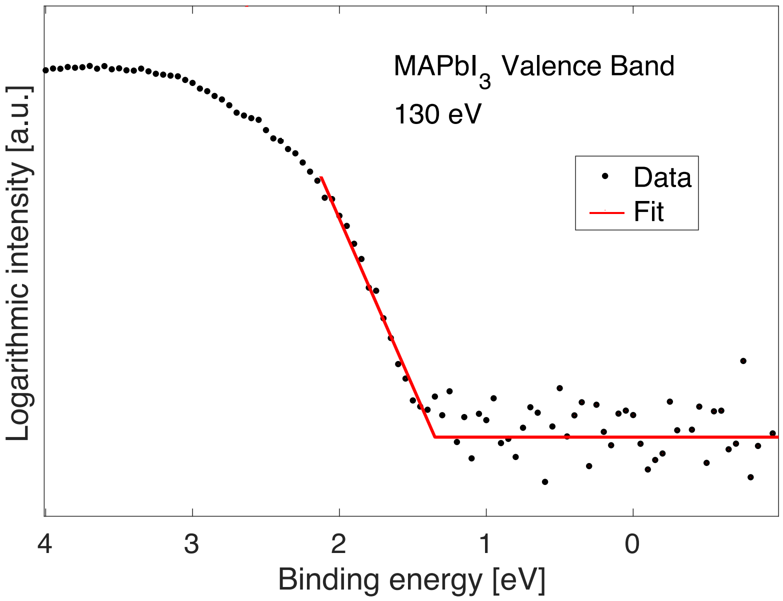

Figure S7: Logarithmic fit valence band $MAPbI_3$. Measured with a photon energy of 130 eV FlexPES beamline. The binding energies were calibrated against Au $4f_{7/2}$.

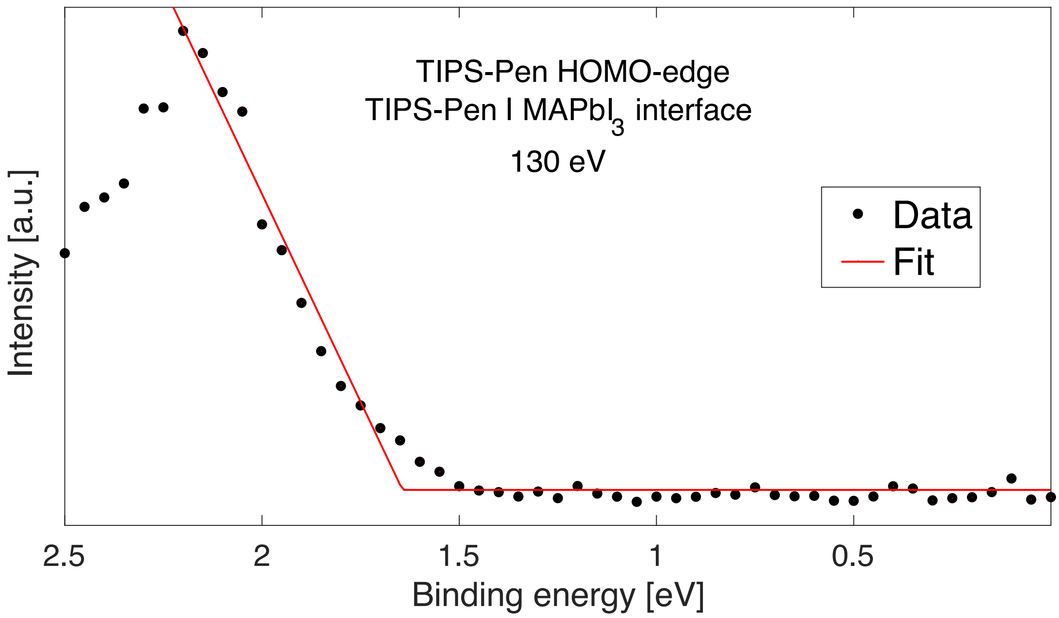

Figure S8: Linear fit of TIPS-Pen HOMO level obtained from the third evaporation of TIPS-Pen on top of $MAPbI_3$ perovskite single crystal. Valence band region measured at 130 eV at FlexPES beamline.

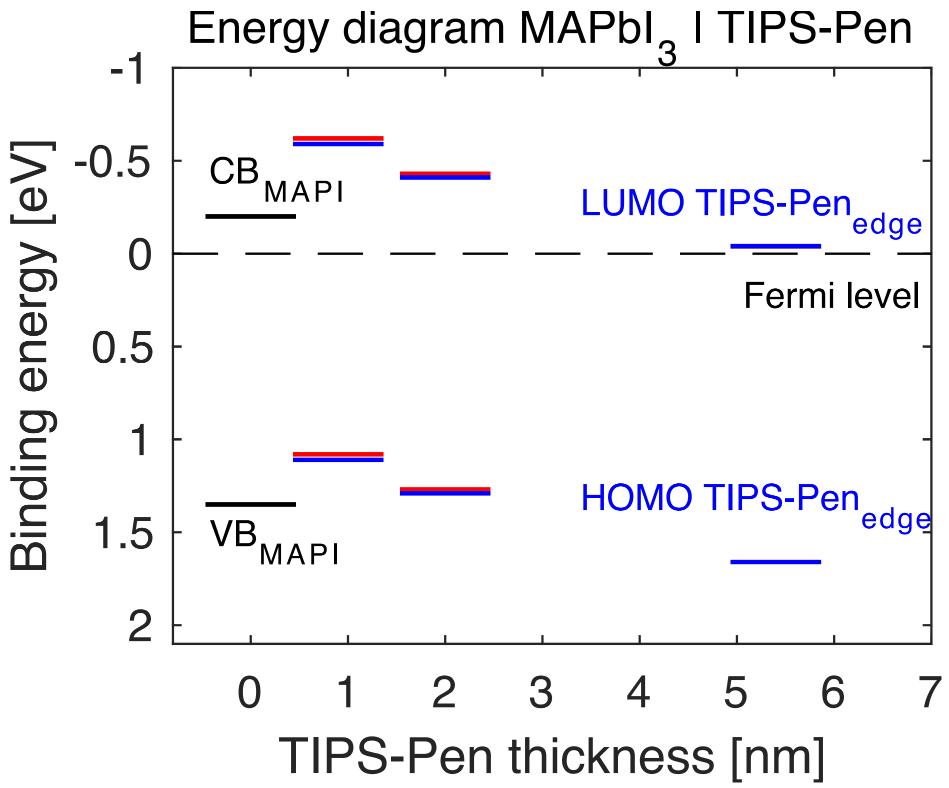

Figure S9: Energy level diagram including $MAPbI_3$ valence band and conduction band (black lines) and summarizing TIPS-Pen HOMO and LUMO band bending obtained by C 1s shifts (blue) and Si 2p shifts (red) The dashed line represents Fermi level.

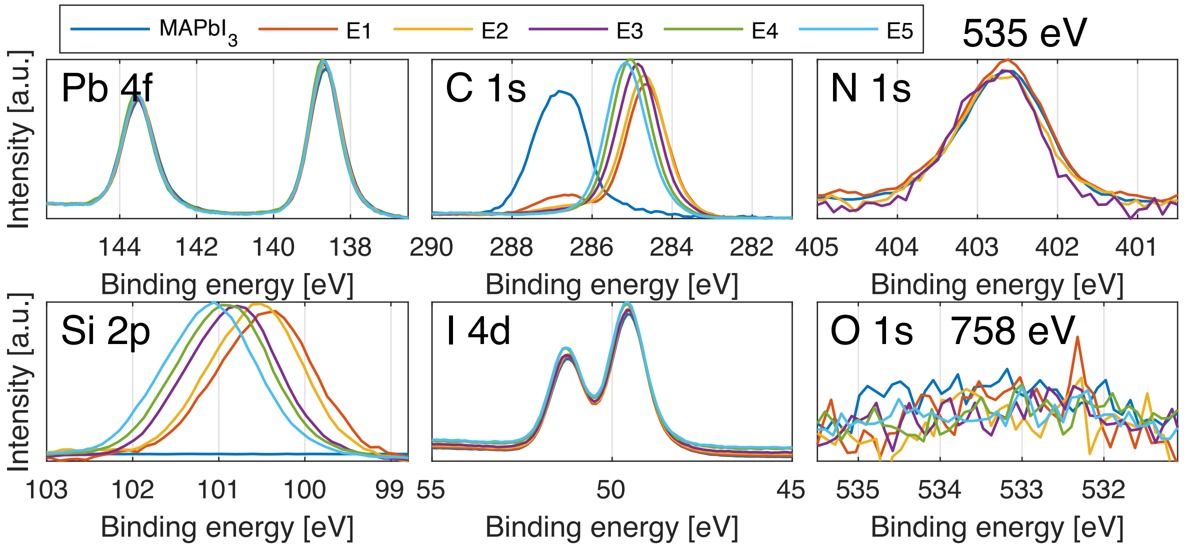
MAPbI3
E1
E2
E3
E4
E5
535 eV
Pb 4f
C 1s
N 1s
Si 2p
I 4d
O 1s
758 eV
Intensity [a.u.]
Binding energy [eV]

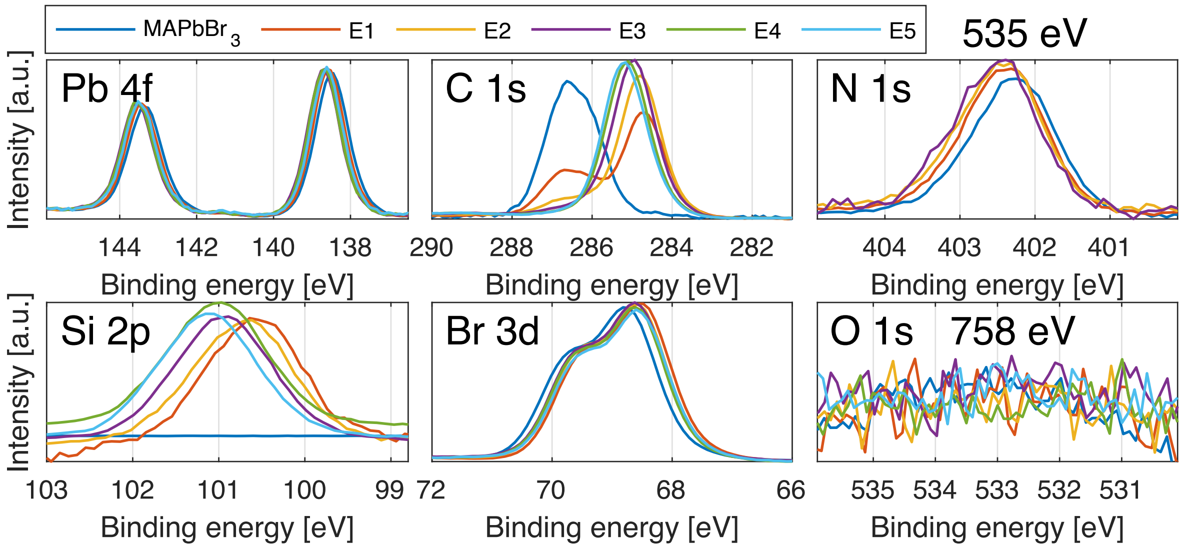
MAPbBr3
E1
E2
E3
E4
E5
535 eV
Pb 4f
C 1s
N 1s
Si 2p
Br 3d
O 1s
758 eV
Intensity [a.u.]
Binding energy [eV]

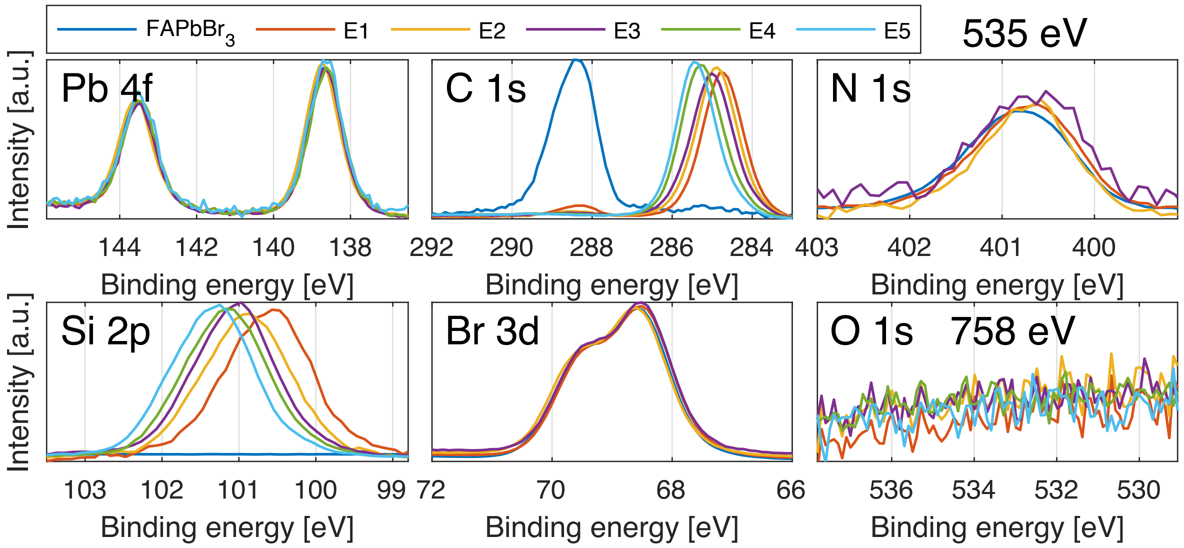
FAPbBr3
E1
E2
E3
E4
E5
535 eV
Pb 4f
C 1s
N 1s
Si 2p
Br 3d
O 1s
758 eV
Intensity [a.u.]
Binding energy [eV]

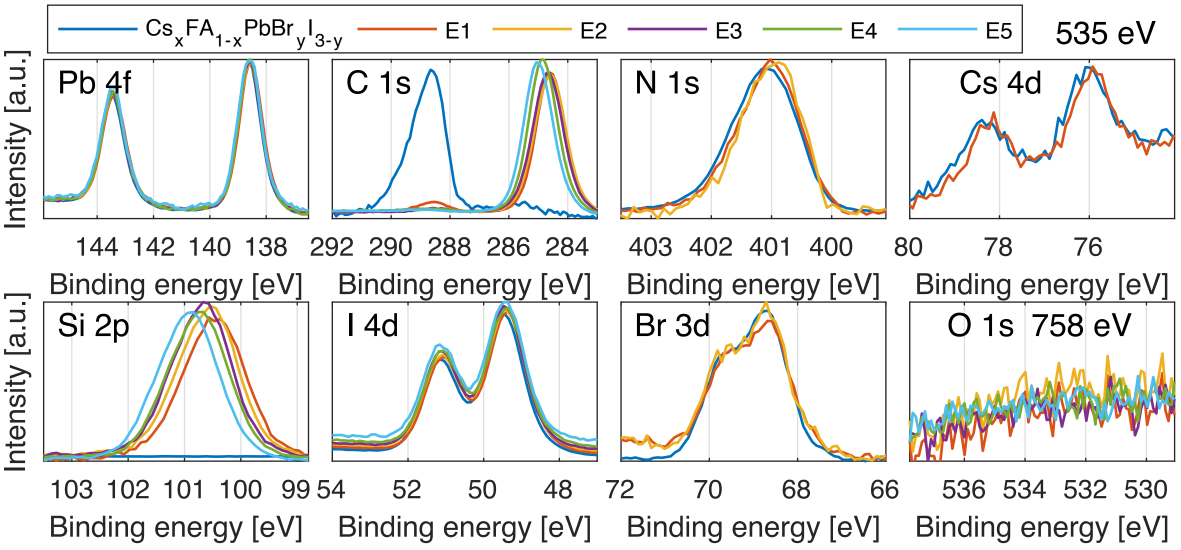

Figure S10: Photoelectron spectra of the Pb 4f, C 1s, N 1s, I 4d, Br 3d, Cs 4d and Si 2p core levels recorded using 535 eV and O 1s using 758 eV from $MAPbI_3$, $MAPbBr_3$, $FAPbBr_3$ and $Cs_xFA_{1-x}PbBr_yI_{3-y}$ single crystals cleaved under vacuum (blue line) and after several TIPS-Pen evaporations (other lines). All core levels were measured at CoESCA endstation at the synchrotron Bessy II, Berlin. Binding energies were energy calibrated against Au $4f_{7/2}$ at 84.0 eV.

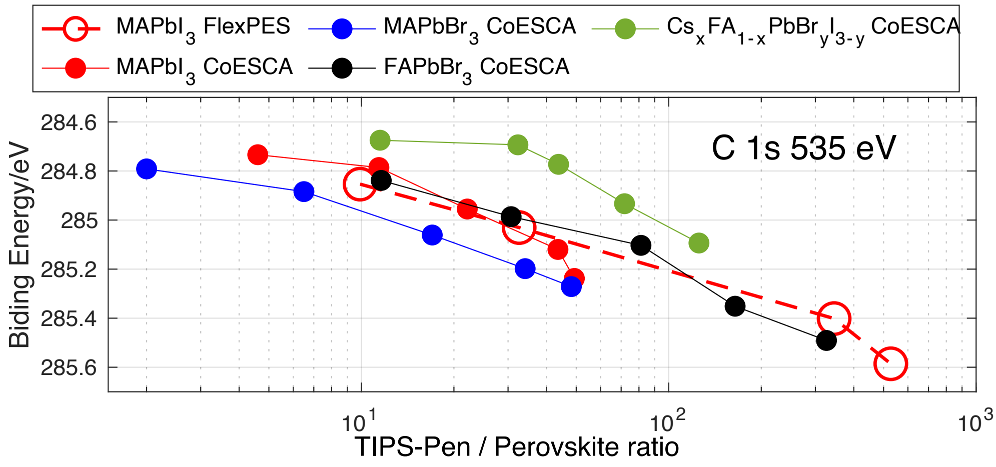

Figure S11: Binding energy shifts of C 1s TIPS-Pen core level after several evaporations on different in-situ cleaved perovskite single crystals. All positions are internally calibrated against Pb 4f core level. Dashed lines represent data obtained from FlexPES beamline and continuous lines represent data obtained at CoESCA endstation.
